# Cellulase over lactic acid bacteria in enhancing antioxidant capacity of mulberry silage via phenolic release

**DOI:** 10.3389/fmicb.2025.1725406

**Published:** 2025-12-11

**Authors:** Yaya Guo, Hongmei Peng, Hailiang Wang, Shu Li, Jiantao Zhao, Wenju Zhang

**Affiliations:** College of Animal Science and Technology, Shihezi University, Shihezi, China

**Keywords:** mulberry silage, lactic acid bacteria, antioxidant capacity, cellulase, phenolic

## Abstract

Mulberry (*Morus alba* L.) is an economically valuable tree rich in phenolics, but its silage quality is limited by low epiphytic lactic acid bacteria (LAB) and water-soluble carbohydrate (WSC) content. This study evaluated the effects of adding epiphytic LAB and cellulase on the fermentation quality, bacterial community, metabolite composition, and antioxidant capacity of mulberry silage. A strain of *Lactiplantibacillus plantarum* LP26, with rapid acid production and high acid tolerance, was isolated and used as an inoculant. Four treatments were applied: untreated control (CK), LAB addition (LAB), cellulase addition (C), and their combination (C_LAB). The results showed that, compared with the CK group, the C and C_LAB groups significantly reduced pH and ammonia nitrogen content, increased lactic acid and WSC, and inhibited yeast and coliforms. They also decreased neutral detergent fiber (NDF), hemicellulose, and cellulose contents. Antioxidant assays showed notably higher DPPH and ABTS radical scavenging activities in the C and C_LAB groups. 16S rRNA sequencing revealed reduced bacterial diversity and increased *Lactobacillus* abundance (96.51%) and decrease *Enterobacter* abundance (5.12%) in C_LAB. Metabolomics analysis indicated that both C and C_LAB markedly altered phenylpropanoid and polyketide profiles, upregulating antioxidants like Camelliaside B and Quercetin 3-O-xylosyl-rutinoside, and enriching pathways such as flavonoid biosynthesis. In conclusion, cellulase enhanced antioxidant capacity by degrading fiber to release phenolics and improve fermentable substrates, while LAB alone had minimal effects. Although combining LAB and cellulase synergistically improved the microbial community, it did not provide additive benefits in phenolic conversion or antioxidant enhancement.

## 1 Introduction

The mulberry tree (*Morus alba* L.), which originated in China, is a highly adaptable multipurpose economic species cultivated worldwide (Fatima et al., 2024). Its leaves contain crude protein at levels comparable to alfalfa hay and exhibit high digestibility, making them a superior woody forage resource. In recent years, phenolic compounds abundant in mulberry leaves and stems have attracted growing interest due to their bioactive properties, such as antioxidant, anti-inflammatory and antimicrobial effects, suggesting promising applications in the food, pharmaceutical and cosmetic industries (Hu et al., 2021; Guo et al., 2024). Studies have indicated that incorporating mulberry silage in place of alfalfa silage can improve antioxidant status and immune function in lambs (Wang and Luo, 2021). Ensiling is a common method used to preserve the nutritional quality of mulberry forage. However, natural ensiling often results in inefficient fermentation and slow acidification, leading to poor silage quality. These limitations are mainly attributed to the low population of epiphytic lactic acid bacteria (LAB) and the limited content of water-soluble carbohydrates (WSC) in fresh mulberry biomass (He et al., 2020). To address these issues, the addition of exogenous LAB or cellulase has been shown to enhance the fermentation process: the former increases the initial LAB count, while the latter breaks down cellulose into fermentable sugars, thus promoting lactic acid (AC) production (Zhao et al., 2018). Although recent research has focused on improving conventional fermentation parameters such as pH and organic acid profiles by adding LAB or cellulase, little attention has been paid to how phenolic compounds change during prolonged ensiling and how these changes affect the antioxidant capacity of the final forage. A systematic understanding of the patterns of phenolic metabolism and their regulatory effects on antioxidant capacity throughout the entire silage process is still lacking.

LAB and cellulase are recognized as safe, efficient and environmentally friendly biological agents that are widely applied in the food sector to enhance the bioactivity of phenolic compounds. These compounds occur in plant tissues mainly in two forms: Free and bound. Free phenolics are stored in cell vacuoles and are readily available for metabolism, whereas bound phenolics are covalently tethered to cell-wall constituents such as cellulose and hemicellulose, which greatly limits their bioaccessibility (Cerda et al., 2013; Yadav et al., 2013). These bound phenolics require the action of gut microbiota or exogenous enzymes, for example through hydrolysis or methylation, to release bioactive constituents (Gonzales et al., 2016; Patil et al., 2024). In fermentation systems, cellulase treatment and microbial metabolism can specifically degrade cell wall structures or hydrolyze glycosidic and ester bonds, thereby markedly enhancing the bioactivity of phenolic compounds. Cellulase processing directly liberates bound phenolics through the hydrolysis of structural polysaccharides such as cellulose and hemicellulose. For instance, cellulase treatment was shown to significantly increase phenolic yield in rice bran (Martillanes et al., 2021), while enzyme complex treatment led to elevated total phenolic content and antioxidant activity in grape pomace (Stanek-Wandzel et al., 2024). LAB fermentation utilizes intracellular enzymes like β-glucosidase and esterase to specifically hydrolyze phenolic glycosides, converting low-activity glycosides into highly active aglycones. This process enhances both antioxidant capacity and bioavailability. For example, A notable increase in flavonoid aglycones was observed in soybeans fermented by *Bifidobacterium* (Peirotén et al., 2020), along with improved sensory properties of the product (Huynh et al., 2014; Peirotén et al., 2020; del Razola-Díaz et al., 2025). However, most existing mechanistic studies have focused on short-term fermentation processes (typically < 7 days) in food applications. There remains a lack of systematic investigation into the transformation patterns of phenolic compounds and the regulation of bioactivity during long-term ensiling processes exceeding 30 days.

Therefore, this study investigated mulberry silage over a 60-day period by adding epiphytic LAB and cellulase. Using 16S rRNA sequencing and untargeted metabolomics, this study addressed two questions: (1) How do LAB and cellulase affect fermentation quality, microbial community, and the composition of metabolites in mulberry silage? (2) Do these additives operate through distinct mechanisms to enhance antioxidant capacity? By integrating fermentation parameters, microbial dynamics, and metabolites profiles, we provide a theoretical basis and practical guidelines for producing mulberry silage with elevated antioxidant activity.

## 2 Materials and methods

### 2.1 Isolation and identification of epiphytic lactic acid bacteria

#### 2.1.1 Silage preparation for isolation of lactic acid bacteria

Mulberry trees with a height of approximately 120 cm were harvested in Kokdala City, Ili Kazakh Autonomous Prefecture, Xinjiang, China (40°14′16″–49°10′45″ N, 80°09′42″–91°01′45″ E), leaving a stubble height of 10 cm. The harvested branches and leaves were wilted in shade for 2 h and then chopped into approximately 2 cm segments. Fermentation was carried out in one-way valve-equipped polyethylene bags (35 × 45 cm). Each bag was filled with 1 kg of material, vacuum-sealed, and incubated at 25°C in darkness for 30 days to facilitate subsequent LAB isolation.

#### 2.1.2 Isolation of epiphytic lactic acid bacteria

A 10 g silage sample was homogenized with 90 mL of sterile saline (0.9% NaCl, Beyotime Biotechnology) and incubated with shaking at 180 r/min and 37°C for 1 h. After filtration, serial dilutions ranging from 10^−1^ to 10^−8^ were prepared and plated onto de Man, Rogosa and Sharpe Agar (MRS) supplemented with calcium carbonate (0.5 g/100 mL, Tianjin Damao Chemical Reagent Factory). Colonies showing distinct calcium dissolution zones (transparent zone diameter ≥ 2 mm), negative catalase activity, and Gram-positive staining were selected and subjected to three rounds of purification. The purified strains were subsequently enriched in MRS broth (Qingdao High-tech Industrial Park Haibo Biotechnology Co., Ltd.). The bacterial suspensions were aliquoted into two parts: one was mixed with an equal volume of glycerol (Xilong Scientific Co., Ltd.) and stored at –80°C, while the other was used for pH measurement. The preserved strains were revived from –80°C storage and subjected to physiological and biochemical characterization following the protocol reported previously (Zhang et al., 2022). Evaluations included microscopic morphology, gas production from glucose, acid production ability, and utilization of 18 carbon sources (such as glucose and lactose). Inoculation was carried out using bacterial micro-biochemical test tubes (Qingdao High-tech Industrial Park Haibo Biotechnology Co., Ltd.), followed by incubation at 37°C for 24–48 h. Carbon source utilization was interpreted based on color changes according to the manufacturer’s protocol.

#### 2.1.3 16 S rDNA sequencing

A 1% bacterial suspension was inoculated into liquid medium (pH 6.2) and cultured at 37°C with shaking at 180 r/min for 24 h. The pH of each culture was measured to preliminarily screen LAB strains with strong acid-producing capability for subsequent sequencing. Genomic DNA was extracted and amplified by PCR using a commercial kit (Beijing TransGen Biotech Co., Ltd.) with primers FA-27F (5′-GCAGAGTTCTCGGAGTCACGAAGAGTTTGATCCTGGCTCA G-3′) and RA-1495 (5′-AGCGGATCACTTCACACAGGACTACG GGTACCTTGTACGA-3′). The 50 μL PCR reaction system comprised 5 μL DNA template, 1 μL of each forward and reverse primer, 25 μL of 2 × Master Mix, and ddH_2_O to volume. The PCR protocol included initial denaturation at 95°C for 10 min; 30 cycles of denaturation at 95°C for 30 s, annealing at 60°C for 30 s, and extension at 72°C for 45 s; with a final extension at 72°C for 5 min. The PCR products were sequenced by Riboxinuke Biotechnology Co., Ltd., and the resulting sequences were compared via BLAST in the NCBI GenBank database.^1^

#### 2.1.4 Tolerance of epiphytic lactic acid bacteria to temperature, acid, and salt stress

The tolerance of the bacterial strains to temperature, acid, and salt was evaluated by inoculating activated cultures into liquid medium under the following conditions: (1) Temperature tolerance: incubation at 5°C for 10 days, 25°C for 3 days, 35°C for 3 days, 40°C for 7 days, and 45°C for 7 days. (2) Acid tolerance: growth at pH levels of 3.0, 3.5, 4.0, 5.0, 6.0, 7.0, and 8.0 for 48 h; (3) Salt tolerance: cultivation in media containing 3.0% (w/v) and 6.5% (w/v) NaCl (Xilong Scientific Co., Ltd) for 48 h.

All cultures were incubated with shaking at 180 r/min, with three biological replicates for each treatment. Following incubation, the optical density at 600 nm (OD_600_) was measured using a visible spectrophotometer (Shanghai Yidian Analytical Instruments Co., Ltd.). The relative growth rate was calculated as a percentage of the OD_600_ under non-stress conditions, which was set as 100%.

#### 2.1.5 Growth and acid production capacity of epiphytic lactic acid bacteria

The LAB selected through physiological, biochemical, and molecular identification were further evaluated for their growth capacity and acid-producing ability by measuring optical density (OD_600_) and pH every 2 h over a 24-h period (0, 2, 4, …, 24 h). pH was measured using a glass-electrode pH meter, and OD_600_ values were determined with a visible spectrophotometer (Shanghai Yidian Analytical Instruments Co., Ltd.).

### 2.2 Silage preparation

The mulberry had a height of approximately 120 cm and were mechanically harvested, leaving a stubble height of 25 cm. After cutting the fresh material into 2 cm segments, 500 g samples were packed into polyethylene silage bags (35 × 45 cm). Four treatment groups were established: (1) CK: blank control; (2) LAB: inoculated with the selected epiphytic *Lactiplantibacillus plantarum* (LP26) at 1 × 10^6^ CFU/g fresh weight (FW); (3) C: supplemented with cellulase at 3 g/kg FW; (4) C_LAB: co-inoculated with 1 × 10^6^ CFU/g FW LAB and 3 g/kg cellulase.

All treatments were supplemented with an equal volume of sterile water, sealed, and ensiled at room temperature for 60 days. After opening the bags, samples were collected for chemical analysis, 16S rRNA sequencing, and plant metabolomic profiling.

### 2.3 Silage fermentation characteristics and antioxidant capacity

Fresh mulberry and silage samples were dried at 65°C to constant weight to determine dry matter (DM) content, then ground and passed through a 0.425 mm sieve. Crude protein (CP) was calculated as Kjeldahl nitrogen × 6.25. Neutral detergent fiber (NDF), acid detergent fiber (ADF), acid detergent lignin (ADL), hemicellulose, and cellulose were determined using the method described by Robertson and Van Soest (Robertson and Van Soest, 1981). WSC were measured using the anthrone method (Arthur Thomas, 1977).

A 10 g sample was homogenized with 90 mL of sterile water (1:9, w/v), filtered through four layers of cheesecloth, and centrifuged at 15,000 × g for 10 min. The supernatant was used for analyzing pH, organic acids, ammonia nitrogen (AN), antioxidant capacity, and microbial counts. pH was measured directly at 25°C using a PHS-3C electrode (Shanghai Yidian Scientific Instrument Co., Ltd.). Organic acids (lactic acid, acetic acid, propionic acid, and butyric acid) were quantified by high-performance liquid chromatography (HPLC) (Ren et al., 2020). AN was determined according to the anthrone colorimetric method described by published report (Weatherburn, 1967). Antioxidant capacity was evaluated using the ferric reducing antioxidant power (FRAP) assay, 2,2’-Azinobis (3-ethylbenzothiazoline-6-sulfonic acid) (ABTS) radical scavenging assay, and 2,2-Diphenyl-1-picrylhydrazyl (DPPH) radical scavenging assay with Griess reagent kits.

For microbial enumeration, the filtrate was serially diluted and plated onto MRS agar for LAB, Yeast Extract-Peptone-Dextrose (YPD) agar for yeasts, and Violet Red Bile Agar (VRBA) for coliforms. Incubation and counting were performed following Wang et al. (Wang et al., 2018).

### 2.4 Bacterial community analysis of mulberry silage

A 20 g silage sample was mixed with 180 mL of pre-chilled 0.85% sterile saline solution and shaken at 4°C for 30 min. The mixture was filtered through four layers of cheesecloth, and the filtrate was centrifuged at 13,000 r/min and 4°C for 10 min. The supernatant was discarded, and the pellet was collected for DNA extraction. Genomic DNA was extracted using a Bacterial DNA Extraction Kit (Yeasen Biotechnology, Shanghai, China) strictly following the manufacturer’s instructions.

The V3–V4 region of the 16S rDNA was amplified using the primers 338F (5’-ACTCCTACGGGAGGCAGCAG-3’) and 806R (5’-GGACTACHVGGGTWTCTAAT-3’). PCR products were examined by 2% agarose gel electrophoresis, purified using magnetic beads, and quantified enzymatically. Equimolar amounts of the amplicons were pooled and subjected to a second electrophoresis. Target bands were recovered using a gel extraction kit.

The constructed libraries were sequenced on the Illumina MiSeq PE250 platform. Raw data were processed through quality control, OTU clustering, and subsequent analyses on the Novogene Magic Platform, with reference to the pipeline described by published report (Tahir et al., 2023). The relevant sequencing data have been deposited in the NCBI database (BioProject: PRJNA1328713).

### 2.5 Metabolomic analysis

#### 2.5.1 Sample preparation

Metabolites from mulberry samples were extracted using a low-temperature methanol-based method. Specifically, 100.0 mg of sample was accurately weighed and mixed with 80% methanol solution pre-chilled to –20°C. Cell disruption was performed through cryogenic grinding at –10°C (50 Hz, 6 min) followed by ultrasonication at 5°C (40 kHz, 30 min). Proteins were precipitated at –20°C for 30 min, and the supernatant was obtained by high-speed centrifugation at 4°C (13,000 × g, 15 min). Quality control (QC) samples were prepared by pooling aliquots of all individual supernatants. Metabolic profiling was carried out using a UHPLC-Q Exactive HF-X system for chromatographic separation and high-resolution mass spectrometric analysis.

#### 2.5.2 Chromatographic conditions

Metabolite extracts were analyzed using an ultra-high-performance liquid chromatography-orbitrap high-resolution mass spectrometry (UHPLC-Orbitrap HRMS) system. Chromatographic separation was carried out on a Waters ACQUITY UPLC HSS T3 column (100 × 2.1 mm, 1.8 μm) with the column temperature set at 40°C, the autosampler maintained at 4°C, and an injection volume of 3 μL. The mobile phase comprised 0.1% formic acid in water (phase A) and 0.1% formic acid in acetonitrile–isopropanol (1:1, v/v, phase B), delivered at a flow rate of 0.40 mL ⋅min^−1^. For positive ion mode, the gradient program was as follows: 0–3.0 min (20% B), 3.0–4.5 min (20–35% B), 4.5–5.0 min (35–100% B), 5.0–6.3 min (100% B), 6.3–6.4 min (100–0% B), and 6.4–8.0 min (0% B). For negative ion mode, the gradient program was: 0–1.5 min (5% B), 1.5–4.5 min (5–30% B), 4.5–5.0 min (30–100% B), 5.0–6.3 min (100% B), 6.3–6.4 min (100–0% B), and 6.4–8.0 min (0% B).

#### 2.5.3 Mass spectrometry conditions

Mass spectrometric detection was performed on a Q Exactive HF-X mass spectrometer equipped with an electrospray ionization (ESI) source. Data were acquired in both positive and negative ion modes. Full MS scans were conducted at a resolution of 120,000 (@ m/z 200) with a scan range of m/z 70–1,050. Data-dependent acquisition (DDA) mode was applied to fragment the top 10 most intense ions from each full scan (dd-MS^2^), with the MS^2^ resolution set to 30,000.

### 2.6 Data analysis

The chemical composition, microbial counts, and antioxidant capacity data of silage samples were analyzed using one-way ANOVA in SPSS Statistics (Version 26.0), with a significance threshold set at *P* < 0.05. Bacterial community and metabolomics data were processed and analyzed on the Majorbio Cloud Platform.

## 3 Results

### 3.1 Physiological and biochemical identification of strains

**FIGURE 1:**
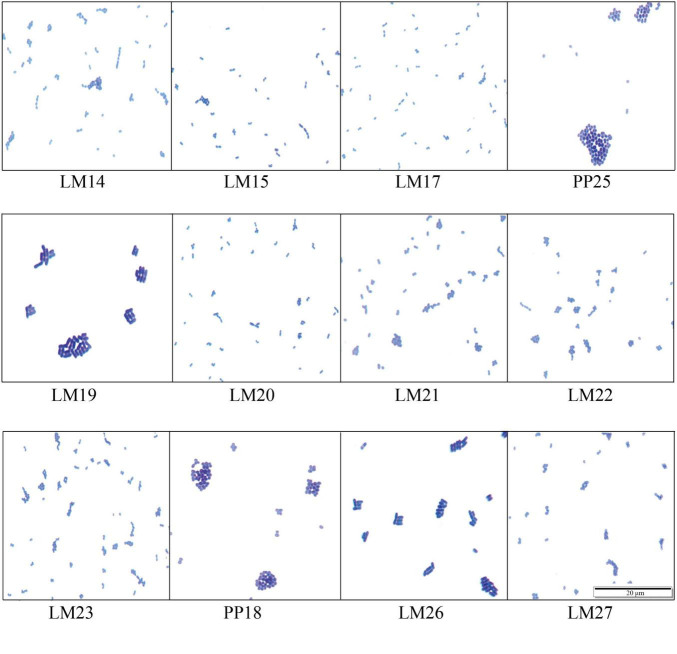
Cellular morphology of the strains.

A total of 12 LAB strains were isolated from mulberry silage after 30 days of natural fermentation. All isolates were Gram-positive, catalase-negative, and capable of acid production (Table 1). Microscopic observations revealed that eight strains exhibited an ovoid morphology and often occurred in pairs; two exhibited spherical shapes, and two were short rods (Figure 1).

**TABLE 1:** Physiological and biochemical characteristics of the strains.

Strains	Gram stain	Catalase	Morphology	Acid production	Gas production
LM14	+	–	Ovoid	+	+
LM15	+	–	Ovoid	+	+
LM17	+	–	Ovoid	+	+
PP25	+	–	Coccus	+	+
LP19	+	–	Bacillus	+	–
LM20	+	–	Ovoid	+	+
LM21	+	–	Ovoid	+	+
LM22	+	–	Ovoid	+	+
LM23	+	–	Ovoid	+	+
PP18	+	–	Coccus	+	+
LP26	+	–	Bacillus	+	–
LM27	+	–	Ovoid	+	+

“+” means positive, “–” means negative.

The utilization of 18 carbon sources by the 12 strains is shown in Table 2. None of the strains could utilize rhamnose; LM14, LM15, and LM27 could not utilize mannose; LM15, LM17, and LM27 could not utilize cellobiose; and LM20, LM21, LM22, and LM23 could not utilize salicin. The remaining carbon sources were utilized by all 12 strains.

**TABLE 2:** Utilization of 18 carbon sources by the strains.

Carbon source	LM 14	LM 15	LM 17	PP 25	LP 19	LM 20	LM 21	LM 22	LM 23	PP 18	LP 26	LM 27
Glucose	+	+	+	+	+	+	+	+	+	+	+	+
Lactose	+	+	+	+	+	+	+	+	+	+	+	+
Xylose	+	+	+	+	+	+	+	+	+	+	+	+
Maltose	+	+	+	+	+	+	+	+	+	+	+	+
Raffinose	+	+	+	+	+	+	+	+	+	+	+	+
Sucrose	+	+	+	+	+	+	+	+	+	+	+	+
Mannose	–	–	+	+	+	+	+	+	+	+	+	–
Inulin	+	+	+	+	+	+	+	+	+	+	+	+
Sorbitol	+	+	+	+	+	+	+	+	+	+	+	+
Rhamnose	–	–	–	–	–	–	–	–	–	–	–	–
Melezitose	+	+	+	–	+	+	+	+	+	–	+	+
Cellobiose	+	–	–	+	+	+	+	+	+	+	+	–
Esculin	+	+	+	+	+	+	+	+	+	+	+	+
Melibiose	+	+	+	+	+	+	+	+	+	+	+	+
Fructose	+	+	+	+	+	+	+	+	+	+	+	+
Salicin	+	+	+	+	+	–	–	–	–	+	+	+
Trehalose	+	+	+	+	+	+	+	+	+	+	+	+
Mannitol	+	+	+	+	+	+	+	+	+	+	+	+

“+” means available; “–” means unavailable.

### 3.2 16 S rDNA identification of strains

BLAST comparison indicated that all 12 isolated strains exhibited over 99% sequence similarity to reference strains. Specifically, eight strains were identified as *Leuconostoc mesenteroides*, two as *Lactiplantibacillus plantarum*, and two as *Pediococcus pentosaceus*, based on 16S rRNA gene sequence homology (Table 3).

**TABLE 3:** Comparison results of 16S rDNA gene sequences of the strains.

Strains	Related species	Similarity
LM14	*Leuconostoc mesenteroides* strain	100.00%
LM15	*Leuconostoc mesenteroides* strain	99.93%
LM17	*Leuconostoc mesenteroides* strain 8m-9	100.00%
PP25	*Pediococcus pentosaceus* strain IAH_35	99.86%
LP19	*Lactiplantibacillus plantarum* strain Sourdough_B11	99.86%
LM20	*Leuconostoc mesenteroides* strain TMPC 33122	99.93%
LM21	*Leuconostoc mesenteroides* subsp. jonggajibkimchii strain 3591	99.93%
LM22	*Leuconostoc mesenteroides* subsp.	100.00%
LM23	*Leuconostoc mesenteroides* subsp.	100.00%
PP18	*Pediococcus pentosaceus* strain Pp-33	99.79%
LP26	*Lactiplantibacillus plantarum* strain TMPC 31113	100.00%
LM27	*Leuconostoc mesenteroides* subsp. jonggajibkimchii strain 3774	99.93%

### 3.3 Growth characteristics of strains

Temperature tolerance varied among strains (Table 4), LM15 and LM17 grew at 5°C, while ten strains exhibited weak growth at 5°C. LM22, LM23, and LM27 grew at 25°C, whereas the other nine strains showed good growth at this temperature. All 12 strains grew well at 35°C and exhibited growth at 40°C. At 45°C, six strains demonstrated weak growth, while the remaining six strains grew.

**TABLE 4:** Tolerance of the strains to temperature, acid, and salt.

Items		LM 14	LM 15	LM 17	PP 25	LP 19	LM 20	LM 21	LM 22	LM 23	PP 18	LP 26	LM 27
Temperature	5 °C	W	+	+	W	W	W	W	W	W	W	W	W
	25 °C	++	++	++	++	++	++	++	+	+	++	++	+
	35 °C	++	++	++	++	++	++	++	++	++	++	++	++
	40 °C	+	+	+	+	+	+	+	+	+	+	+	+
	45 °C	W	W	+	+	W	+	+	W	W	+	+	W
pH	3.0	–	–	–	W	–	–	–	–	–	W	–	–
	3.5	+	–	–	+	W	–	–	–	–	+	+	–
	4.0	+	+	+	+	+	+	+	w	w	+	+	W
	5.0	++	++	++	++	++	++	++	+	+	++	++	+
	6.0	++	++	++	++	++	++	++	+	+	++	++	+
	7.0	++	++	++	++	++	++	++	+	+	++	++	+
	8.0	+	+	+	+	+	+	+	+	+	+	+	+
NaCl(%)	3%	+	+	+	+	+	+	+	+	+	+	+	+
	6%	w	w	w	+	+	w	+	w	w	+	w	+

++, Good growth (OD_600_ > 0.8); +, Growth (0.5 < OD_600_ ≤ 0.8); W, Weak growth (0.2 < OD_600_ ≤ 0.5); –, No growth (OD_600_ ≤ 0.2).

In terms of acid tolerance, PP25 and PP18 showed weak growth at pH 3.0, while the other strains did not grow. At pH 3.5, LP19 exhibited weak growth; LM14, PP25, PP18, and LP26 grew. At pH 5.0, pH 6.0, and pH 7.0, strains LM22, LM23, and LM27 grew, while the other strains showed good growth. All strains grew at pH 8.0.

Under salt stress, all 12 strains grew in the presence of 3% NaCl. At 6% NaCl, LM14, LM15, LM17, LM20, LM22, LM23, and LP26 displayed weak growth, while the other five strains grew.

### 3.4 Growth and acid production capacity of the 12 strains

**FIGURE 2:**
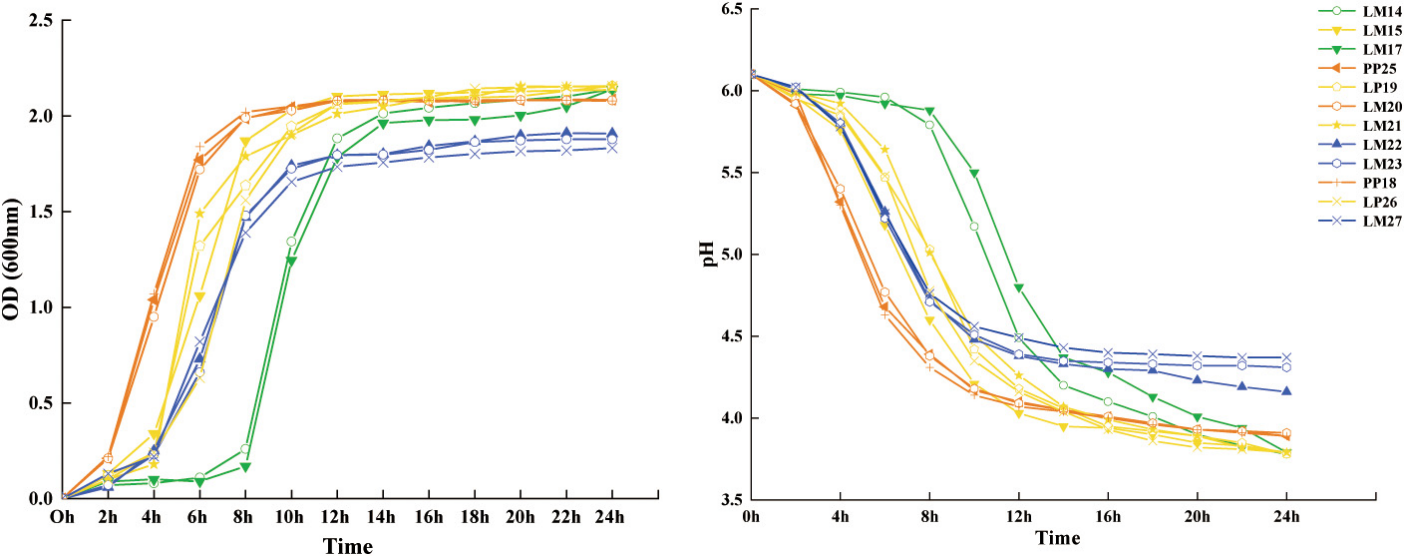
Growth capacity and acid production capacity of strains within 24 h.

The growth patterns of the 12 strains were categorized into four types (Figure 2). Three strains entered the logarithmic phase within 2 h and reached the stationary phase by 12 h, with a final OD_600_ value of 2.08. Seven strains exhibited delayed entry into the logarithmic phase (4 h) and attainment of the stationary phase (16 h). Among these, four strains (LM15, LP19, LM21, and LP26) reached a higher OD_600_ value (2.15), while three strains showed lower OD_600_ values (1.83–1.90). The remaining two strains entered the logarithmic phase at 8 h and reached the stationary phase after 18 h, achieving OD_600_ values similar to the former group.

Acid production trends were closely correlated with growth patterns. After 24 h of cultivation, six strains (LM14, LM17, LM15, LP19, LM21, and LP26) reduced the pH to 3.8, while three strains exhibited poor acidification ability (pH > 4.0). Based on rapid acid production capacity and acid tolerance, strain LP26 was selected as a potential inoculant for subsequent silage trials.

### 3.5 Chemical characteristics and microbial counts of fresh mulberry

The chemical characteristics and microbial counts of fresh mulberry are summarized in Table 5. The pH was 6.72, while the DM and CP contents were 29.76% and 20.05%, respectively. The NDF and ADF contents were 554.42 g/kg DM and 307.63 g/kg DM, respectively. The WSC concentration was 10.02%. The counts LAB, yeasts, and coliforms were 5.68 log_10_ CFU/g FW, 4.51 log_10_ CFU/g FW, and 6.13 log_10_ CFU/g FW, respectively.

**TABLE 5:** Chemical characteristic and microbial counts of fresh mulberry.

Items	Contents
pH	6.72 ± 0.05
Dry matter (%)	29.76 ± 0.52
Crude protein (%)	20.05 ± 0.61
Neutral detergent fiber (g/kg DM)	554.42 ± 10.68
Acid detergent fiber (g/kg DM)	307.63 ± 4.29
Water soluble carbohydrate (%)	10.02 ± 0.43
LAB (log_10_ CFU/g FW)	5.68 ± 0.45
Yeast (log_10_ CFU/g FW)	4.51 ± 0.28
Coliform (log_10_ CFU/g FW)	6.13 ± 0.13

### 3.6 Chemical characteristics and microbial counts of mulberry silage

As shown in Table 6, no significant differences were observed in DM or CP content between the treatment groups and the control group. The C and C_LAB groups exhibited similar fermentation characteristics, with pH values (4.23 and 4.15, respectively) being significantly lower than those of the CK (4.91) and LAB (4.75) groups (*P* < 0.01), along with higher LA contents (49.14 and 41.72%, respectively). The C group had the highest WSC content (7.67%, *P* < 0.01), while the C_LAB group showed the lowest AN content (0.24 g/kg, *P* < 0.01). Regarding organic acids, the AC content in the LAB group (23.30 g/kg DM) was significantly higher than that in the CK group (*P* < 0.05), and the PA content in the CK group (1.58 g/kg DM) was significantly higher than those in all treatment groups (*P* < 0.01). Butyric acid (BA) was not detected in any group. Microbiological analysis revealed that the LAB count was the highest in the C group (7.37 log_10_ CFU/g), while the counts of yeast and coliform bacteria in the CK group were significantly higher than those in the other groups.

**TABLE 6:** Chemical characteristic and microbial population of mulberry silage.

Items	Treatments				SEM	*P*
	CK	C	LAB	C_LAB		
DM (%)	29.25 ± 0.52	30.01 ± 0.20	29.48 ± 0.22	29.51 ± 0.35	0.084	0.063
pH	4.91 ± 0.17^a^	4.23 ± 0.02^b^	4.75 ± 0.04^a^	4.15 ± 0.05^b^	0.032	< 0.01
CP (% DM)	17.40 ± 1.05	18.45 ± 0.45	17.21 ± 0.67	19.99 ± 1.75	0.388	0.116
AN (g/kg FW)	0.52 ± 0.04^a^	0.31 ± 0.02^c^	0.45 ± 0.02^b^	0.24 ± 0.02^d^	0.010	< 0.01
WSC (% DM)	5.79 ± 0.20^b^	7.67 ± 0.57^a^	5.52 ± 0.64^b^	5.44 ± 0.34^b^	0.167	< 0.01
LA (g/kg DM)	25.48 ± 0.78^b^	49.14 ± 1.16^a^	28.19 ± 6.87^b^	41.72 ± 1.91^a^	1.285	< 0.01
AC (g/kg DM)	18.38 ± 1.35^b^	19.68 ± 069^ab^	23.30 ± 2.35^a^	22.00 ± 0.90^ab^	0.517	< 0.05
PA (g/kg DM)	1.58 ± 0.20^a^	0.19 ± 0.07^b^	0.04 ± 0.01^b^	0.04 ± 0.00^b^	0.037	< 0.01
BA (g/kg DM)	ND	ND	ND	ND	–	-
LAB (log_10_ CFU/g FW)	6.11 ± 0.23^b^	7.37 ± 0.28^a^	6.31 ± 0.18^b^	7.05 ± 0.43^b^	0.104	< 0.01
Yeast (log_10_ CFU/g FW)	3.98 ± 0.17^a^	2.84 ± 0.05^b^	2.67 ± 0.44^b^	2.91 ± 0.38^b^	0.108	< 0.05
Coliform (log_10_ CFU/g FW)	4.56 ± 0.14	3.83 ± 0.20	3.22 ± 0.11	< 2	–	–

Values sharing the same superscript letter within a row are not significantly different. Values with different superscript letters within a row are significantly different.

As shown in Table 7, significant differences were observed in the structural carbohydrate composition among the treatment groups. Compared with the CK group (549.63 g/kg DM), the NDF content was significantly reduced in the C and C_LAB groups (504.97 g/kg DM and 513.20 g/kg DM, respectively; *P* < 0.01). The hemicellulose content was lowest in the C group (195.45 g/kg DM), being highly significantly lower than that in all other groups. The C_LAB group (208.66 g/kg DM) was also significantly lower than the LAB and CK groups (*P* < 0.01). The cellulose content in the C and C_LAB groups (267.46 g/kg DM and 265.94 g/kg DM, respectively) was highly significantly lower than that in the CK and LAB groups. No significant differences were detected in ADF or ADL contents among all treatments.

**TABLE 7:** Structural carbohydrates of mulberry silage.

Items	Treatments				SEM	*P*
	CK	C	LAB	C_LAB		
NDF (g/kg DM)	549.63 ± 5.70^a^	504.97 ± 5.55^b^	541.62 ± 7.32^a^	513.20 ± 4.44^b^	2.066	< 0.01
ADF (g/kg DM)	318.55 ± 4.99	307.90 ± 5.76	310.75 ± 4.53	304.54 ± 4.22	2.050	0.499
ADL (g/kg DM)	40.55 ± 6.17	42.07 ± 4.24	37.95 ± 3.91	38.59 ± 2.40	1.553	0.781
Hemicellulose (g/kg DM)	235.97 ± 3.71^a^	195.45 ± 3.90^c^	230.87 ± 7.20^a^	208.66 ± 5.45^b^	1.859	< 0.01
Cellulose (g/kg DM)	273.11 ± 1.86^a^	267.46 ± 1.61^b^	272.79 ± 1.47^a^	265.94 ± 1.98^b^	0.616	< 0.01

Values sharing the same superscript letter within a row are not significantly different. Values with different superscript letters within a row are significantly different.

### 3.7 Antioxidant capacity of mulberry

**FIGURE 3:**
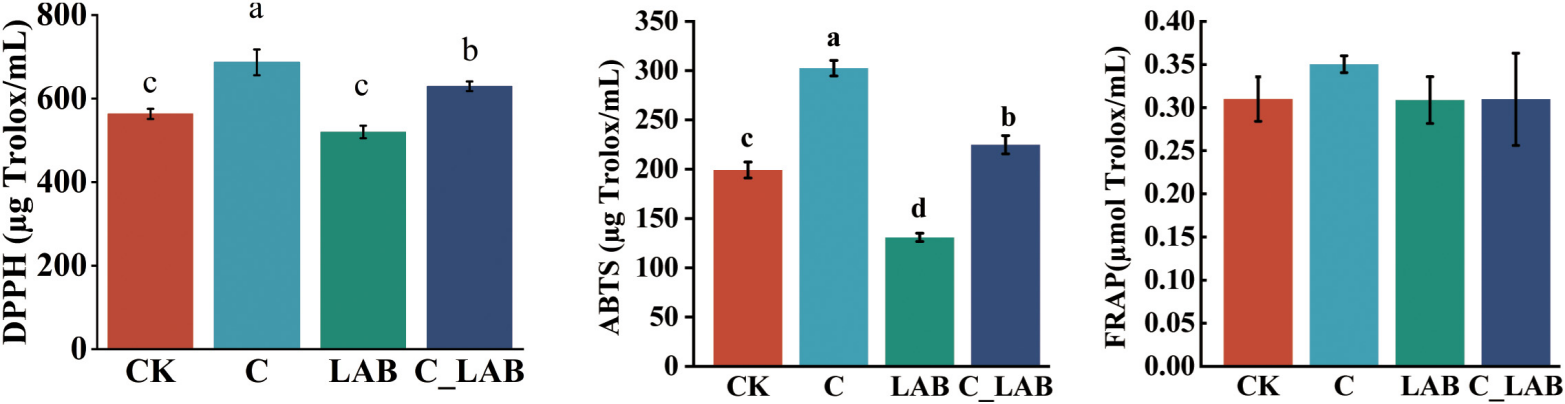
Antioxidant capacity of mulberry silage (the same letters indicate no statistically significant difference, while different letters denote a statistically significant difference).

The antioxidant capacity assay results are shown in Figure 3. The C group exhibited the highest DPPH (686.92 μg Trolox/mL) and ABTS (302.45 μg Trolox/mL) scavenging capacities, which were significantly higher than all other groups (*P* < 0.01). The C_LAB group (DPPH: 629.45 μg Trolox/mL; ABTS: 224.86 μg Trolox/mL) was also significantly higher than the CK and LAB groups (*P* < 0.01). No significant differences in FRAP levels were observed among the groups.

### 3.8 Bacterial community characteristics of mulberry silage

**FIGURE 4:**
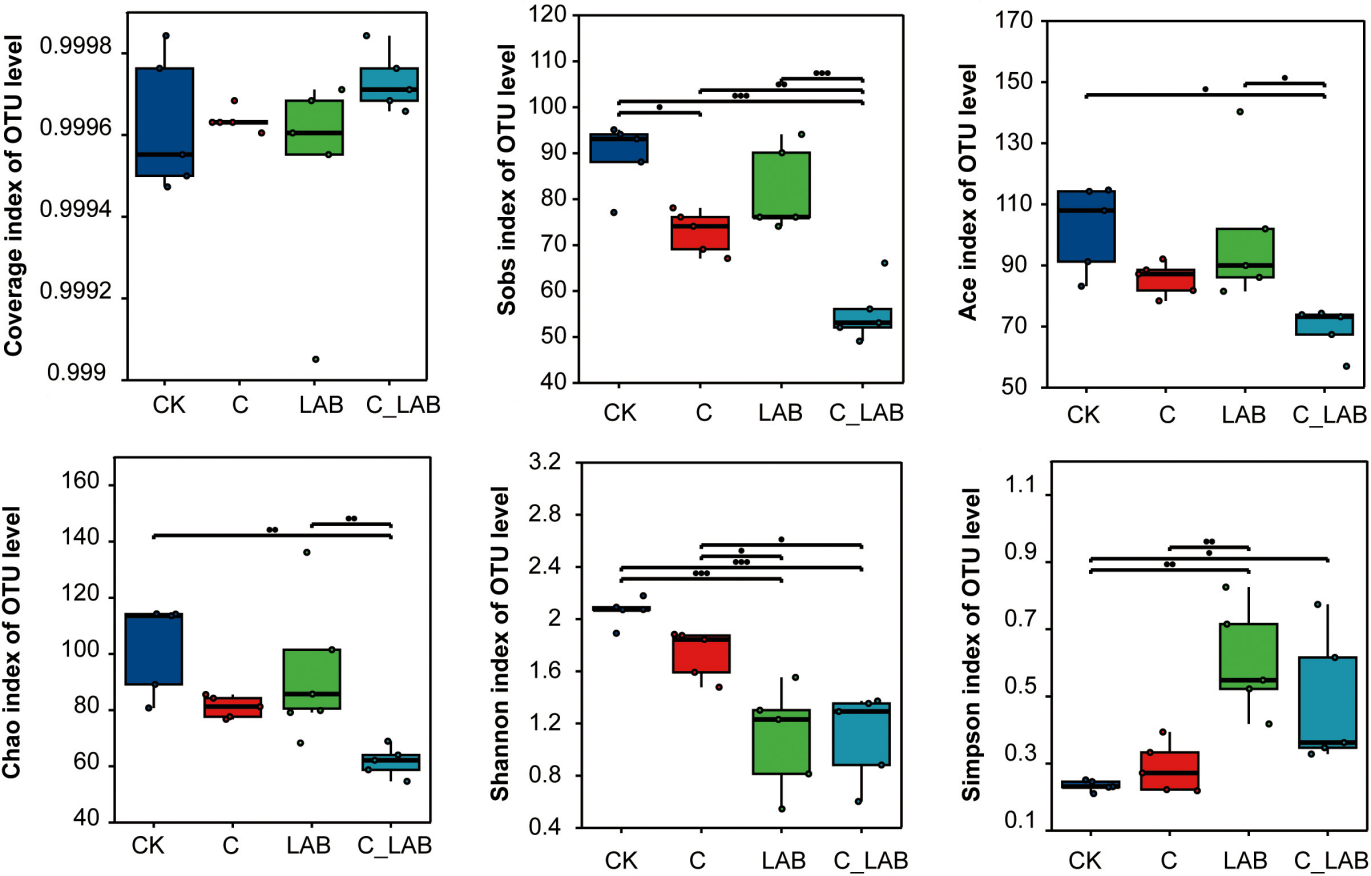
Alpha diversity analysis of the bacterial community in mulberry silage. *P < 0.05, **P < 0.01, ***P < 0.001.

Analysis of bacterial communities after 60 days of ensiling revealed significant differences in alpha diversity among treatment groups (Figure 4). In terms of community richness, the C_LAB group showed significantly lower values in the Sobs (55.20), ACE (69.00), and Chao (61.55) indices compared to the CK group (Sobs: 89.40; ACE: 102.10; Chao: 102.23) and the LAB group (Sobs: 82.00; ACE: 99.82; Chao: 96.46) (*P* < 0.05). Regarding community diversity, both the LAB (1.09) and C_LAB (1.10) groups exhibited significantly lower Shannon indices than the CK (2.06) and C (1.73) groups (*P* < 0.05), while the Simpson index demonstrated an opposite trend, with significantly higher values in the LAB (0.60) and C_LAB (0.48) groups compared to the CK (0.23) and C (0.29) groups (*P <* 0.05). The sequencing coverage of all groups approached 1 (range: 0.99952–0.99973), confirming adequate sequencing depth and reliable results.

**FIGURE 5:**
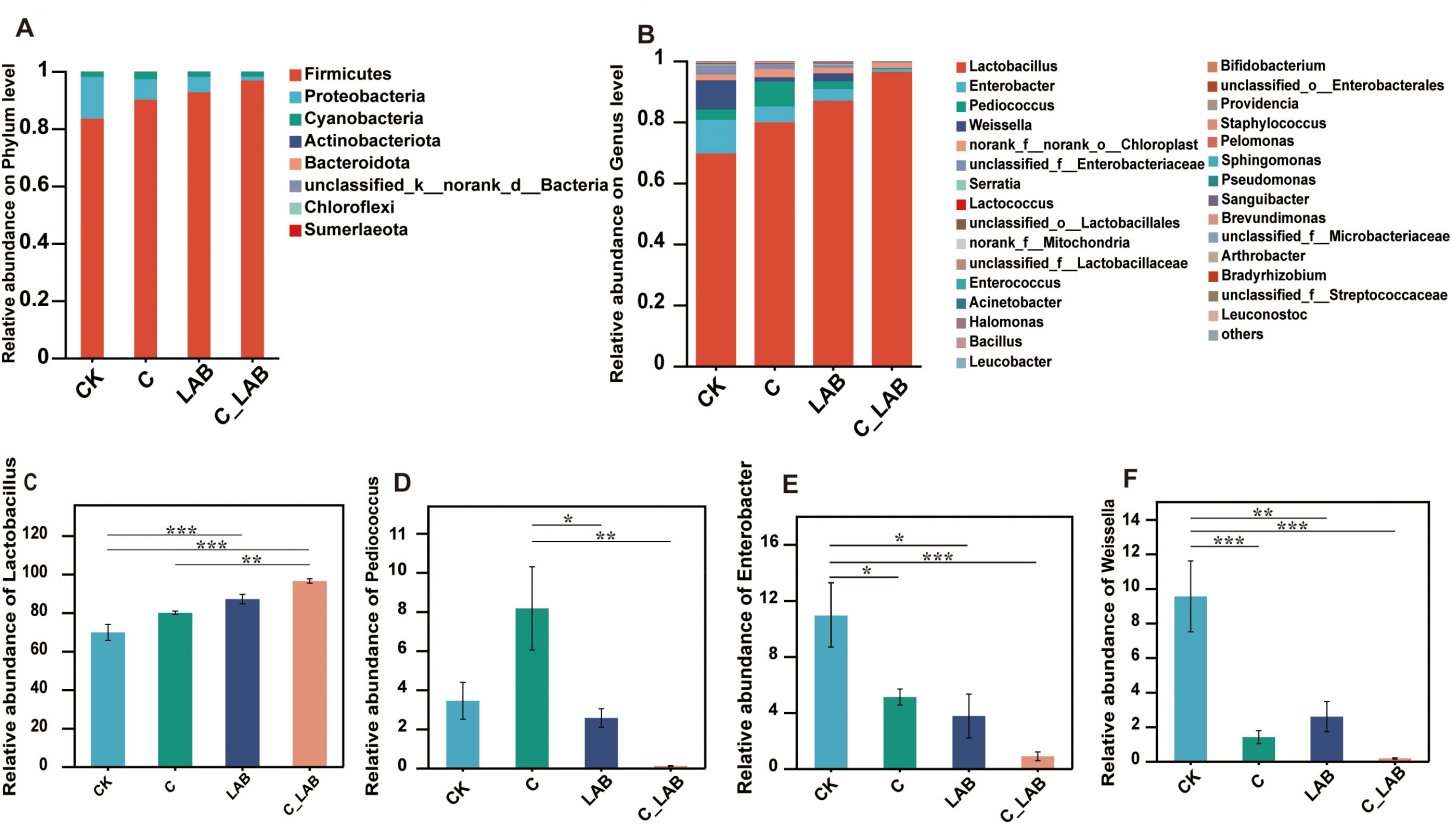
Bacterial community of mulberry silage on phylum level (A) and on genus level (B); relative abundance of *Lactobacillus* (C), *Pediococcus* (D), *Enterobacter* (E), *Weissella* (F). *P* < 0.05, *P < 0.01, **P < 0.001.

Fermentation treatments significantly altered the bacterial community composition at the phylum level (Figure 5A). The CK group was dominated by Firmicutes (83.58%), followed by Proteobacteria (14.54%) and Cyanobacteria (1.81%). Compared to the CK group, the relative abundance of Firmicutes significantly increased in the C, the LAB and C_LAB groups (90.18%, 92.76%, and 96.91%, respectively, *P* < 0.01), while that of Proteobacteria significantly decreased in the C, LAB, and C_LAB groups (reduced to 7.14%, 5.32%, and 1.33%, respectively, *P* < 0.01). Additionally, the relative abundance of Cyanobacteria in the C group showed a significant increase to 2.6% (*P* < 0.05). No significant changes were observed in other phyla among the treatment groups.

The genus level composition is shown in Figure 5B. The dominant genus in the CK group was *Lactobacillus* (69.79%), followed by *Enterobacter* (10.94%) and *Weissella* (9.55%) and *Pediococcus* (3.45%). Both LAB and C_LAB groups significantly increased the abundance of *Lactobacillus* (87.07 and 96.51%, respectively, *P* < 0.01, Figure 5C), with the C_LAB group being significantly higher than the C group (80.00%, *P* < 0.01), while no significant difference was found between the CK and C groups. The relative abundance of *Pediococcus* in the C group (8.17%) was significantly higher than that in the LAB (2.57%) and C_LAB (0.12%) groups (*P* < 0.01), while no significant difference was observed when compared with the CK group (3.45%) (Figure 5D). The CK group showed relative abundances of *Enterobacter* and *Weissella* at 10.94% and 9.55%, respectively. All treatment groups demonstrated significantly reduced abundances of *Enterobacter* (0.89%–5.12%; Figure 5E) and *Weissella* (0.18%–2.59%; Figure 5F) compared to the CK group (*P* < 0.01), with the C_LAB group exhibiting the lowest values (0.89 and 0.18%, respectively).

**FIGURE 6:**
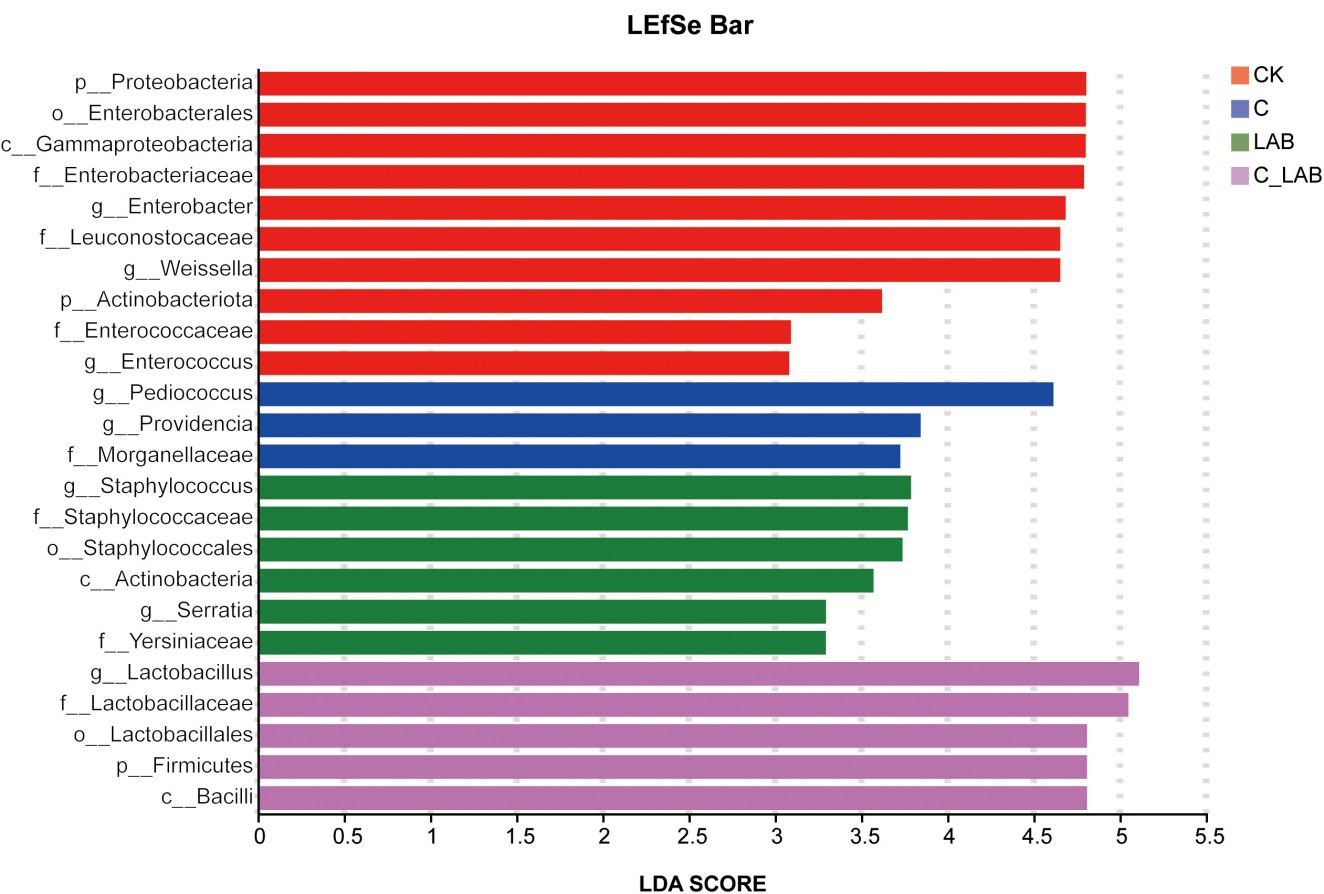
Linear discriminant analysis scores of discriminative microbial taxa identified by LEfSe analysis among different treatment groups.

LEfSe analysis revealed specifically enriched microbial taxa at the genus level in mulberry silage across different treatment groups (Figure 6). The C group was primarily enriched with *Pediococcus* and *Providencia*; the LAB group was specifically enriched with *Staphylococcus*; while the C_LAB group showed significant enrichment of *Lactobacillus*.

**FIGURE 7:**
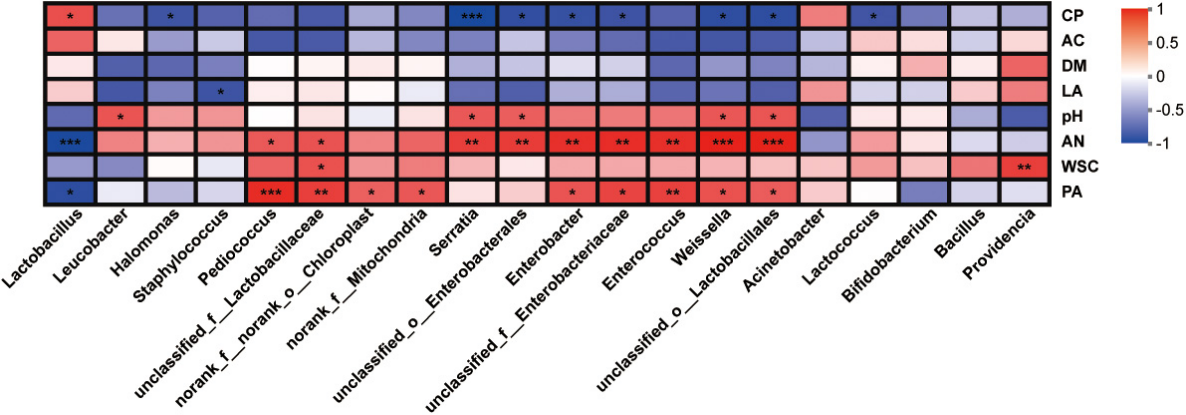
Correlation heatmap between chemical characteristics and the top 20 bacterial genera by relative abundance in mulberry silage. *P < 0.05, **P < 0.01, ***P < 0.001.

The correlations between the top 20 most abundant bacterial genera at the genus level and the fermentation characteristics of mulberry silage are shown in Figure 7. *Lactobacillus* was significantly positively correlated with CP (*R* = 0.65, *P* < 0.05) and highly significantly negatively correlated with AN (*R* = –0.83, *P* < 0.001). *Enterobacter*, *unclassified_f__Enterobacteriaceae*, and *Enterococcus* showed highly significant positive correlations with AN (*R* = 0.80, *P* < 0.01; *R* = 0.81, *P* < 0.01; *R* = 0.80, *P* < 0.01, respectively), while *Enterobacter* and *unclassified_f*__*Enterobacteriaceae* showed significant negative correlations with CP (*R* = –0.63, *P* < 0.05; *R* = –0.60, *P* < 0.05, respectively). *Pediococcus* was significantly positively correlated with AN (*R* = 0.60, *P* < 0.05).

### 3.9 Metabolite profiles

**FIGURE 8:**
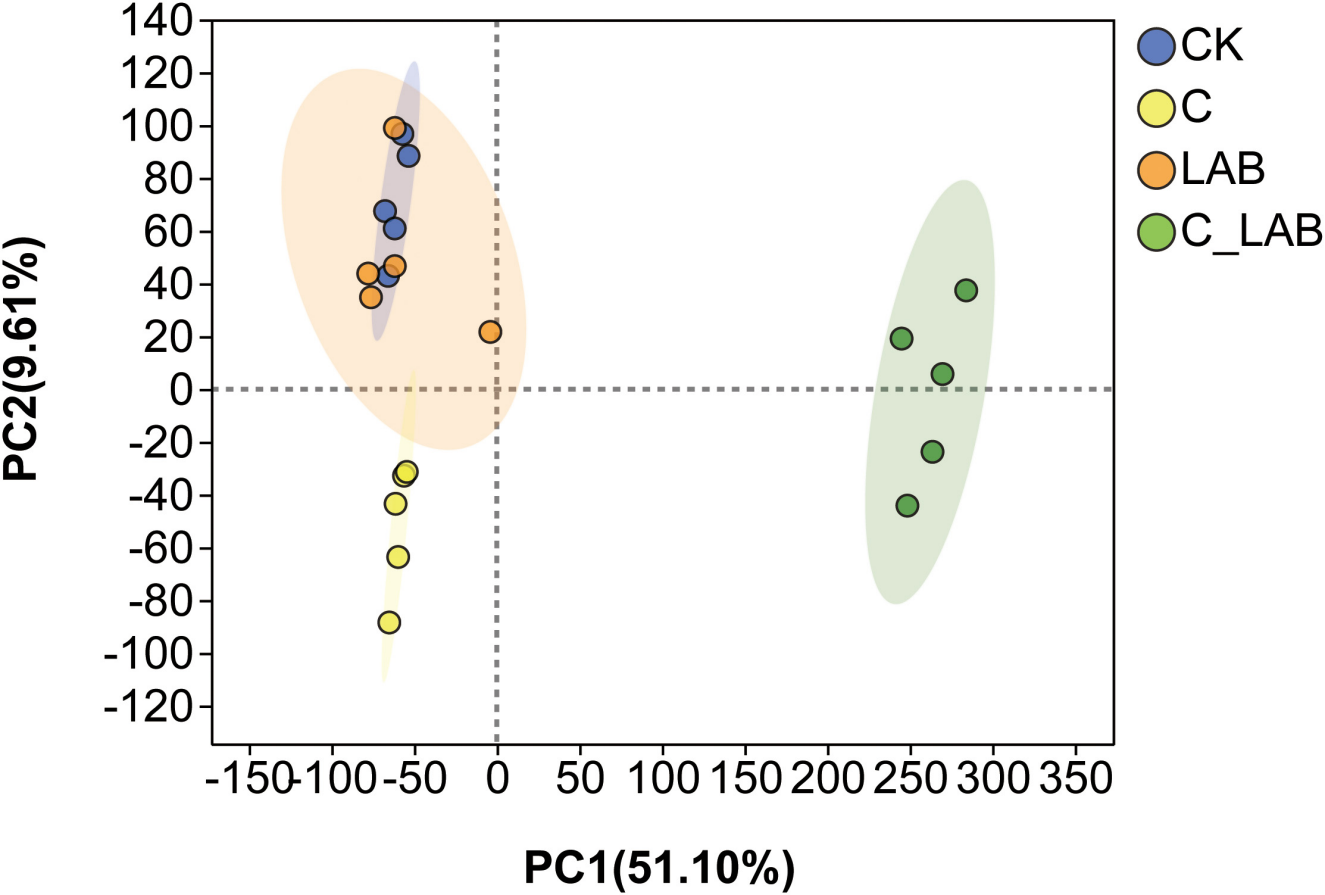
Principal component analysis of metabolite profiles from mulberry silage.

Principal component analysis (PCA) of metabolites from different treatment groups is shown in Figure 8. The cumulative contribution rate of PC1 and PC2 reached 60.71%. The metabolite profiles of the C and C_LAB groups were significantly distinct and separated from those of the CK and LAB groups, while no significant difference was observed between the CK and LAB groups. These results indicate that cellulase treatment alone and its combination with LAB had a greater impact on differentiating metabolite composition than LAB treatment alone.

**FIGURE 9:**
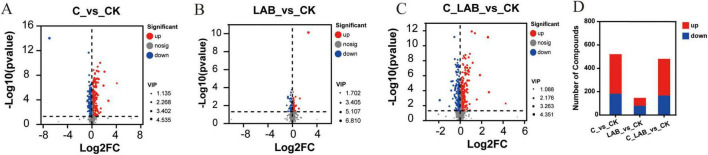
Differential metabolite profiles in mulberry silage treatments. (A) Volcano plot of C vs. CK. (B) Volcano plot of LAB vs. CK. (C) Volcano plot of C_LAB vs. CK. (D) Statistical counts of up- and down-regulated metabolites for each comparison.

Volcano plot analysis further compared differential metabolites between each treatment group and the CK group (Figure 9). A total of 17,934 metabolites were identified, among which 1,325 were differential metabolites. Compared with the CK group, the C group contained 519 significantly differential metabolites (338 up-regulated and 181 down-regulated; *P* < 0.05), the LAB group had 145 (67 up-regulated and 78 down-regulated; *P* < 0.05), and the C_LAB group showed 479 (313 up-regulated and 166 down-regulated; *P* < 0.05).

**FIGURE 10:**
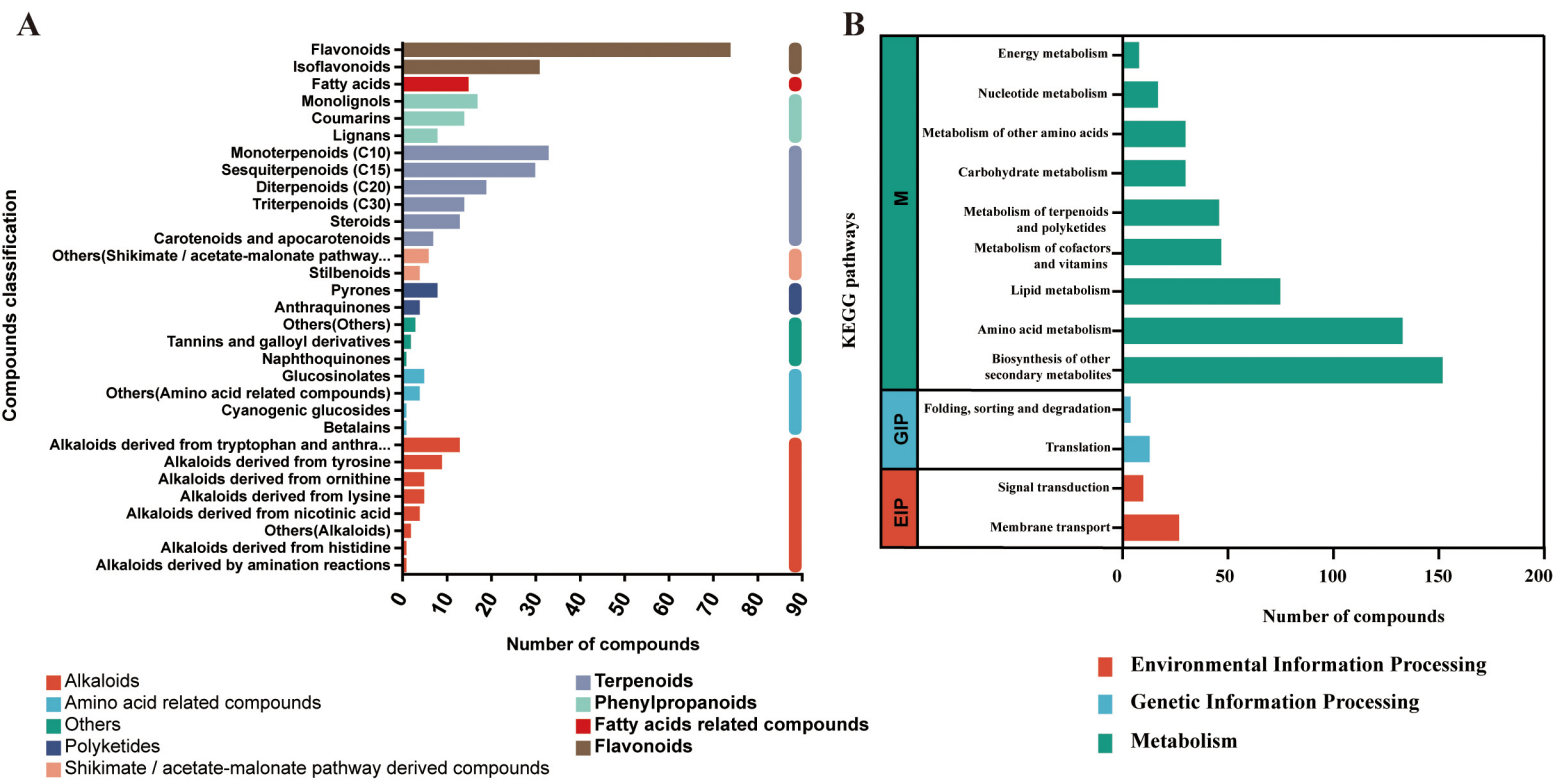
KEGG classification (A) and pathway enrichment analysis (B) of metabolites identified in mulberry silage.

KEGG annotation identified a total of 354 plant secondary metabolites (Figure 10A), with terpenoids (116 compounds) being the most abundant, followed by flavonoids (105) and alkaloids (40). Pathway enrichment analysis (Figure 10B) revealed that 539 compounds were mapped to the “Metabolism” pathway. Further analysis showed that, within the subcategories of the “Metabolism” pathway, the “Biosynthesis of other secondary metabolites” subpathway was enriched with 152 compounds.

**FIGURE 11:**
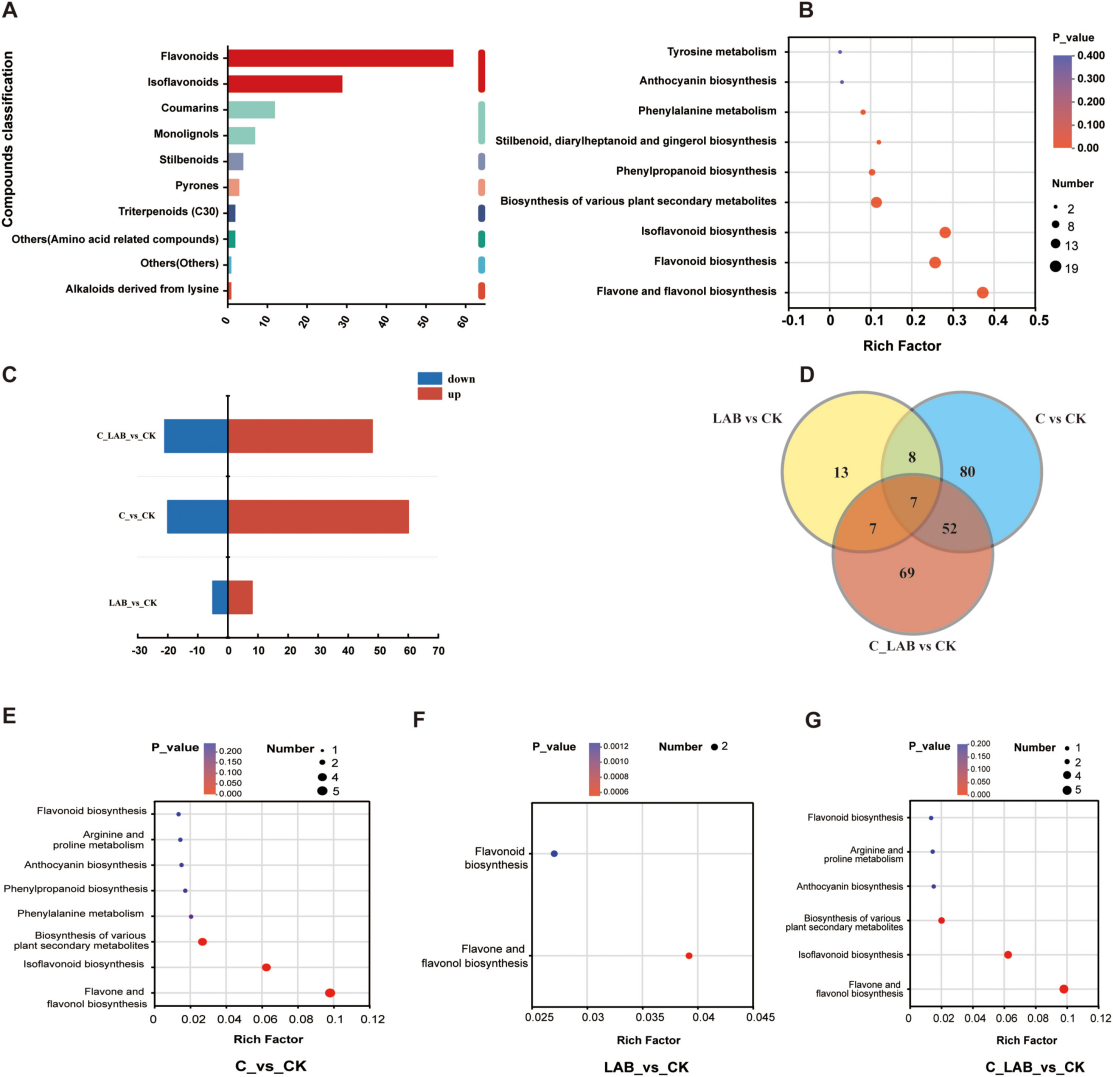
Analysis of phenylpropanoid and polyketide metabolites in mulberry silage based on KEGG database. (A) Composition and classification of identified phenylpropanoids and polyketides. (B) Pathway annotation of all identified phenylpropanoids and polyketides. (C) Number of differential metabolites between treatment groups and the CK group. (D) Venn diagram showing overlaps of differential phenylpropanoids and polyketides among different comparative groups. (E–G) Pathway annotation of differential phenylpropanoids and polyketides in (E) LAB vs. CK, (F) C vs. CK, and (G) C_LAB vs. CK comparisons.

This study investigated changes in metabolites associated with antioxidant capacity in mulberry silage, with a focus on phenylpropanoids and polyketides. KEGG annotation identified 204 compounds (Figure 11A), primarily including flavonoids (57), isoflavonoids (29), and coumarins (12), which were mainly enriched in pathways such as flavonoid and isoflavonoid biosynthesis (Figure 11B).

Differential expression analysis revealed significant differences between each treatment group and the CK group (Figure 11C). The C vs. CK comparison showed the most pronounced differences, with 80 differentially expressed metabolites (60 up-regulated and 20 down-regulated), dominated by flavonoids (28.75%) and cinnamic acid derivatives (17.50%) (Supplementary Figures 1A,B). Subclass level distribution is shown in Supplementary Figures 1A,C. These metabolites were enriched in eight antioxidant-related pathways, such as flavonoid biosynthesis (Figure 11E). The LAB vs. CK comparison had 13 differentially expressed metabolites (8 up-regulated, 5 down-regulated), predominantly flavonoids (76.92%) (Supplementary Figures 2A,B), and was enriched in two pathways (Figure 11F). At the subclass level, flavonoid glycosides were the most abundant (46.14%) (Supplementary Figures 2A,C). The C_LAB vs. CK comparison exhibited 69 differentially expressed metabolites (48 up-regulated, 21 down-regulated), with flavonoids (33.33%) and cinnamic acid derivatives (17.39%) as the major types (Supplementary Figures 3A,B). Subclass-level classification is shown in Supplementary Figures 3A,C. Compared to the C vs. CK comparison, the enriched pathways in the C_LAB vs. CK comparison lacked “phenylpropanoid biosynthesis” and “phenylalanine metabolism” (Figure 11G).

Venn diagram analysis (Figure 11D) revealed that among the significantly differential phenylpropanoids and polyketides, the LAB vs. CK and C vs. CK comparisons shared 8 metabolites, the LAB vs. CK and C_LAB vs. CK comparisons shared 7 metabolites, and the C vs. CK and C_LAB vs. CK comparisons shared 52 metabolites. All three comparisons (LAB vs. CK, C vs. CK, and C_LAB vs. CK) shared 7 metabolites in common.

**FIGURE 12:**
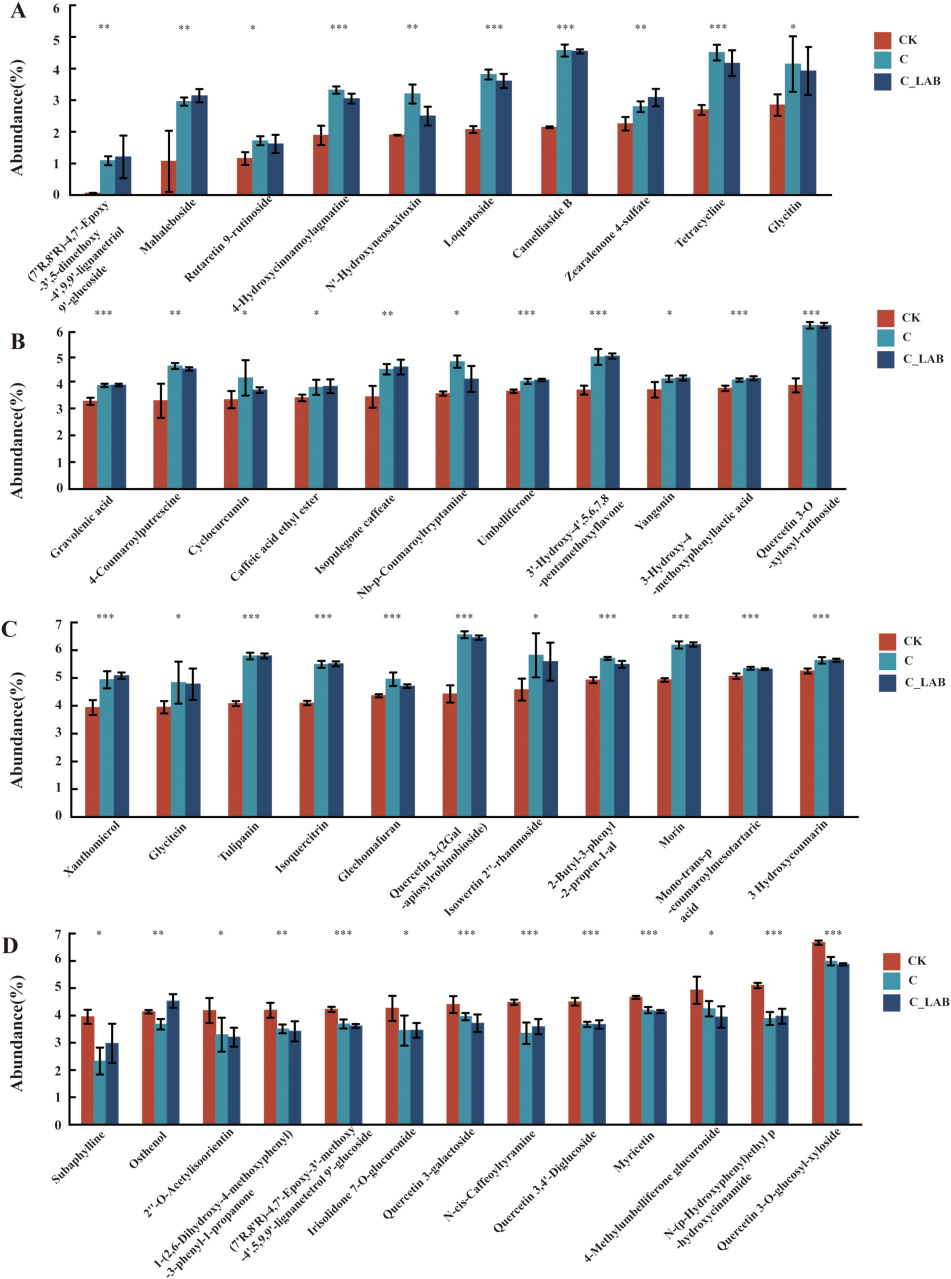
Relative abundance of the 45 common phenylpropanoids and polyketides identified in both the C vs. CK and C_LAB vs. CK comparisons. (A–C) 32 polyketides that were significantly upregulated in both comparative groups; (D) 12 polyketides that were significantly downregulated in both comparative groups (including Osthenol with opposing regulation trends). *P < 0.05, **P < 0.01, ***P < 0.001.

Beyond the seven metabolites shared by all three treatment groups, the C vs. CK and C_LAB vs. CK comparisons exhibited 45 common phenylpropanoids and polyketides. Among these, Osthenol showed opposing regulation trends (down-regulated in C vs. CK and up-regulated in C_LAB vs. CK), while the remaining 44 metabolites demonstrated entirely consistent regulatory directions: 12 were significantly down-regulated (Figure 12D, *P* < 0.05) and 32 were significantly up-regulated (Figures 12A–D, *P* < 0.05). The fold change (FC) values of these metabolites were highly consistent between the two comparative groups, except for (7’R,8’R)-4,7’-Epoxy-3’-methoxy-4’,5,9,9’-lignanetetrol 9’, which showed the largest FC difference (Δ = 1.87). The FC differences of the other 44 metabolites ranged from –0.21 to 0.37, with 34 exhibiting only minor differences (–0.05 to 0.09).

Among the 32 commonly upregulated compounds (Figures 12A–C), Camelliaside B showed the highest VIP values (C vs. CK: 3.27; C_LAB vs. CK: 3.28; Supplementary Table 1). Quercetin 3-O-xylosyl-rutinoside had VIP scores of 3.13 in C vs. CK and 3.15 in C_LAB vs. CK, while Mahaleboside showed a VIP of 2.82 in C_LAB vs. CK. FC analysis indicated that (7’R,8’R)-4,7’-Epoxy-3’,5-dimethoxy-4’,9,9’-lignanetriol 9’-glucoside exhibited the strongest up-regulation (C: 17.17; C_LAB: 19.04), followed by Mahaleboside (C: 2.77; C_LAB: 2.94) and Camelliaside B (C: 2.13; C_LAB: 2.12).

Among the 13 commonly downregulated compounds (Figure 12D), Subaphylline showed the highest VIP value (2.57) in the C vs. CK comparison, while Quercetin 3,4’-Diglucoside exhibited the highest VIP value (2.99) in the C_LAB vs. CK comparison. FC analysis revealed that Quercetin 3-galactoside, Quercetin 3-O-glucosyl-xyloside, and Myricetin all had FC values of 0.90 in the C vs. CK comparison. In the C_LAB vs. CK comparison, Myricetin showed the lowest FC value (0.89), followed by Quercetin 3-O-glucosyl-xyloside (0.88).

## 4 Discussion

### 4.1 Analysis of physiological characteristics of lactic acid bacteria

This study revealed significant inter-strain differences in carbon source utilization and tolerance to temperature, acid, and salt among LAB of the same species. These variations originate from genomic diversity (Lin et al., 2022) and environmental adaptability (Techtmann et al., 2016; Chen et al., 2023). For instance, plant-derived strains metabolize plant carbohydrates more efficiently than dairy-derived strains (Chen et al., 2023), suggesting that epiphytic LAB from mulberry are better adapted to its carbohydrate composition and are therefore more suitable for silage fermentation.

Significant differences in acid production capacity and acid tolerance were observed among different strains. These functional variations may be associated with differences in key enzymes, operon structures, and transport capabilities (Makarova et al., 2006). In terms of acid tolerance, only LP19 and PP18 exhibited minimal growth at pH 3.0. Since the low-pH environment in the later stages of ensiling can inhibit LAB activity (Makarova et al., 2006), acid tolerance is also a critical trait. Based on a comprehensive evaluation of these two core characteristics, namely rapid acid production to reduce initial pH and acid tolerance to adapt to late-stage conditions, LP26 was selected as a candidate inoculant for subsequent mulberry silage trials to further assess its practical efficacy in silage applications.

### 4.2 Chemical characteristics of mulberry silage

In this study, the C and C_LAB groups significantly improved the fermentation quality of mulberry silage, with a notable reduction in pH (C group: 4.23; C_LAB group: 4.15). Although slightly above the ideal range (3.8–4.2) (Du et al., 2022), these values were significantly lower than that of the CK group (4.91), indicating that both cellulase treatment alone and its combination with LAB effectively promoted LA fermentation and acidification. A LA/AC ratio > 3 indicates homolactic fermentation dominance, whereas a ratio close to 1:1 suggests heterolactic fermentation dominance (Abdel-Rahman et al., 2011; Blajman et al., 2018). The LA/AC ratios in the C and C_LAB groups were 2.50:1 and 1.89:1, respectively, higher than those in the CK group (1.38:1) and LAB group (1.20:1). This demonstrates that the combined application of cellulase and LAB synergistically increased the proportion of LA, shifting the fermentation pattern toward homolactic fermentation, which is more conducive to silage preservation.

From the perspective of the underlying mechanism, cellulase and LAB exhibit effective functional complementarity in the process of pH reduction. A strictly anaerobic environment serves as a prerequisite for initiating LA fermentation, as it not only suppresses the activity of aerobic microorganisms but also creates favorable conditions for LAB to become the dominant microbial population. Within this system, cellulase significantly increases the content of WSC by degrading cellulose and hemicellulose in the plant cell wall (Sun et al., 2025). This is specifically demonstrated by the significantly higher WSC content in the C group (7.67%) compared to the CK group (5.79%) and the LAB group (5.52%) in this study, thereby providing a more abundant fermentable substrate for the growth and metabolism of LAB (Du et al., 2023). LAB, in turn, efficiently utilizes these substrates to produce large amounts of LA and other organic acids through homofermentative or heterofermentative pathways, directly driving the decrease in system pH (Maneerat et al., 2015). A noteworthy observation is that the WSC content in the C_LAB group (5.44%) was lower than in the C group, which is likely attributable to accelerated substrate consumption resulting from increased LAB population and enhanced metabolic activity (Hassanat et al., 2007). Furthermore, the lower WSC level in the LAB group (5.52%) confirms the limited ability of most LAB strains to degrade structural carbohydrates (Li F. et al., 2020), as they primarily rely on endogenous or enzymatically released WSC for growth (Sun et al., 2025). Although the substrate-degrading action of cellulase and the acid-producing phase of LAB are intertwined during fermentation, making it difficult to strictly delineate their temporal sequence, together they form a mutually reinforcing synergistic cycle: Abundant substrates ensure rapid acid production by LAB and subsequent system acidification, and the resulting low-pH environment further inhibits the growth of undesirable microorganisms (Zanine et al., 2010), thereby maintaining stable microecological conditions for the continued action of cellulase and the successful progression of LA fermentation.

All treatment groups significantly suppressed the growth of yeast and coliform. Exogenous LAB inhibited yeast by rapidly consuming oxygen and creating an anaerobic environment (Du et al., 2023), while the higher acetic acid content in the treatment groups (19.68–23.30 g/kg DM) further enhanced this inhibitory effect. Coliform, as the primary competitors for WSC and major producers of AN (Pahlow et al., 2003; Wang et al., 2022), showed substantially reduced viability (<2 log_10_ CFU/g FW) in the C_LAB group, where the pH decreased to 4.15 (below the inhibition threshold of pH < 4.5 (Huang R. Z. et al., 2022). The reduction in AN content indicated that the combined application of cellulase and LAB effectively inhibited the proteolytic activity of spoilage bacteria represented by coliform, better preserving true protein and thereby improving the nutritional value of the silage (Yuan et al., 2015).

### 4.3 Structural carbohydrates in mulberry silage

In this study, changes in the structural carbohydrate composition of mulberry silage primarily resulted from the addition of exogenous cellulase. Compared with the CK group, treatments involving cellulase (C and C_LAB groups) significantly reduced the NDF content, which can be attributed to the degradation of hemicellulose and cellulose components. Specifically, the most pronounced degradation of hemicellulose was observed in the C group, while cellulose content in both the C and C_LAB groups was significantly lower than that in the CK and LAB groups. Hemicellulose, as the most abundant and structurally diverse polysaccharide in plant cell walls (Agger et al., 2014), is more susceptible to enzymatic hydrolysis than cellulose due to its amorphous structure and lower degree of polymerization (Xia et al., 2024). Therefore, cellulase effectively hydrolyzes cellulose and hemicellulose in the cell wall, disrupting the fiber structure and breaking down part of the insoluble macromolecules into soluble sugars that can be utilized by microorganisms, thereby providing additional substrates for microbial activity (Ma et al., 2025). This result is consistent with the observation in section 3.6 that the C group had the highest WSC content, indicating that sugars released through cellulase action significantly promoted LAB fermentation, enhanced acid production, and further reduced pH.

The results of this study showed no significant difference in NDF and cellulose content between the C and C_LAB groups, and the hemicellulose content in the C group was even lower than that in the C_LAB group. This suggests that the combination of cellulase and LAB did not exhibit a synergistic effect on the degradation of structural carbohydrates. This phenomenon may be related to differences in cellulase activity under varying pH conditions. Previous studies have indicated that some cellulases exhibit stronger degradation capacity for lignocellulose at relatively higher pH levels (Ma et al., 2025). In this study, the pH in the C group was higher than that in the C_LAB group, which may explain the more thorough degradation of hemicellulose in the C group. Similar results were reported in a previous study (He et al., 2025), where the ADF content in the cellulase-alone treatment group was lower than that in the group treated with a combination of cellulase and *Lactiplantibacillus plantarum*, further supporting the important influence of pH on cellulase efficacy.

In this study, the treatment involving only LAB did not significantly affect any fiber components, indicating that LAB alone lack the ability to directly degrade fiber structures. The ADL content remained stable across all treatment groups, with no significant differences observed, as ADL is the most recalcitrant component of lignocellulose during ensiling (Ma et al., 2025).

### 4.4 Antioxidant capacity of mulberry silage

Ensiling treatments significantly enhanced the antioxidant activity of mulberry silage (except the LAB group); the C group showed the highest DPPH and ABTS scavenging, followed by C_LAB, indicating cellulase plays a key role in improving the antioxidant capacity of mulberry silage, whereas sole LP26 had limited effect, consistent with our previous study (Guo et al., 2024).

Cellulase promotes fiber decomposition by degrading cell walls and may also release intracellular bound antioxidants, while providing substrates for fermentation to activate secondary metabolic pathways and enhance the biosynthesis and transformation of antioxidants (Vong et al., 2017). Similar studies have confirmed that enzymatic hydrolysis releases bound phenolic acids. The antioxidant activity of phenolic compounds is related to their molecular structures: DPPH scavenging depends on phenolic hydroxyl groups, while ABTS scavenging is influenced by both phenolic hydroxyl and methoxy groups (Jiang et al., 2024). Studies indicate that 4%–57% of phenolic compounds exist in plant tissues in bound forms via covalent bonds, hydrophobic interactions, or hydrogen bonds with cell wall polysaccharides such as dietary fiber (Huang D. et al., 2022). The enhanced antioxidant capacity observed in the C and C_LAB groups in this study may be primarily attributed to cellulase-mediated degradation of cell walls, which promotes the release of bound phenolic acids (e.g., ferulic acid and gallic acid) and flavonoids, thereby improving free radical scavenging ability.

### 4.5 Bacterial community of mulberry silage

Silage fermentation is inherently a complex process of microbial ecological succession (Yang et al., 2024). The results of this study demonstrate that compared to the CK and C groups, the individual or combined supplementation of LP26 (LAB and C_LAB groups) significantly reduced bacterial community richness and diversity, which represents a typical characteristic of high-quality silage (Yang et al., 2024). These two treatment groups effectively promoted LAB to become the dominant microbiota, resulting in a structurally simplified and functionally specialized microbial community. Specifically, the relative abundances of *Lactobacillus* reached 87.07% to 96.51%, respectively (Figure 5C). In contrast, although cellulase supplementation alone increased the abundance of LAB, it failed to establish a simplified community structure dominated by *Lactobacillus*. These findings indicate that mulberry silage requires additional LAB inoculants to ensure successful fermentation, which aligns with the conclusions of our previous research (Guo et al., 2024).

At the phylum level, Firmicutes was the dominant phylum in all groups, with the highest abundance (96.91%) observed in the C_LAB group. In contrast, Proteobacteria, which is associated with spoilage, was significantly inhibited in all treatment groups, with the strongest suppression in the C_LAB group, demonstrating a clear synergistic effect between the LAB and cellulase in suppressing this phylum. Firmicutes are key microorganisms that thrive in the anaerobic and acidic environment formed during ensilage, participating in the acid hydrolysis of organic matter. Many bacteria within this phylum, particularly lactic acid bacteria (such as *Lactobacillus*), exhibit acid tolerance (Li W. et al., 2020). This aligns with our isolation results: all 12 screened LAB strains exhibited varying growth capacity at pH 4.0. The resulting low-pH environment selectively inhibits the growth of pH-sensitive spoilage bacteria (such as *Enterobacter* within the Proteobacteria phylum). These harmful microorganisms compete with LAB for fermentable substrates, thereby increasing the risk of spoilage in silage. Studies have demonstrated that the activity of *Enterobacter* is significantly inhibited when the pH falls below 4.7 (Su et al., 2019). Consequently, the acidic environment creates a competitive advantage for acid-tolerant Firmicutes, enabling them to proliferate extensively and dominate the microbial community (Wang et al., 2023).

Genus level analysis revealed that although *Lactobacillus* (69.79%) was dominant in the CK group, relatively high proportions of *Enterobacter* (10.94%) and *Weissella* (9.55%) were also present. All treatments significantly increased the abundance of *Lactobacillus* and reduced the abundance of *Enterobacter*, with the most pronounced effects observed in the C_LAB group. This indicates that LAB and cellulase promote the proliferation of *Lactobacillus* and inhibit harmful bacteria through different pathways, exhibiting a synergistic effect. Specifically, the addition of LAB alone directly increased the initial viable count of LAB at the beginning of fermentation (Gou et al., 2025), while the addition of cellulase alone degraded cell walls to release WSC (Du et al., 2023), providing substrates for LAB growth. In contrast, the combined treatment (C_LAB) simultaneously supplemented bacterial inoculum and enhanced substrate availability, further optimizing the microbial community structure through synergistic effects. In the C_LAB group, the significant increase in *Lactobacillus* and enhanced substrate supply, coupled with a sharp decline in *Enterobacter*, collectively resulted in markedly reduced pH and AN content. This shift is critical, as it indicates suppressed protein degradation, thereby better preserving the nutritional value of the silage.

As a representative of homofermentative LAB (Cai et al., 1999), *Lactobacillus* efficiently converts WSC into LA, rapidly reducing pH and inhibiting harmful microorganisms (Wang et al., 2025), thereby reducing protein degradation and AN production. Correlation analysis showed that *Lactobacillus* was significantly positively correlated with CP and highly significantly negatively correlated with AN. The high abundance of *Enterobacter* (10.94%) in the CK group and its significant positive correlation with AN may explain the higher AN accumulation in this group. *Pediococcus* in the C group contributed to rapid acid production (Ke et al., 2025). However, it is noteworthy that *Pediococcus pentosaceus* showed a significant positive correlation with AN (consistent with previous findings (Guo et al., 2024)), possibly because it enhances the degradation activity of *Enterobacter* toward amino acids (Huang R. Z. et al., 2022). This conclusion, however, requires further experimental validation.

### 4.6 Enhanced antioxidant capacity in mulberry silage by cellulase via phenolic release

PCA of metabolites revealed clear separation between the metabolic profiles of the C and C_LAB groups compared to the CK and LAB groups, indicating that cellulase serves as the core factor reshaping the metabolic profile. Although both C_LAB and LAB groups outperformed the C and CK groups in reducing microbial diversity and increasing the relative abundance of LAB, the metabolic profile of the LAB group highly overlapped with that of the CK group, with no significant improvement in antioxidant capacity. This demonstrates that the individual addition of L*actobacillus plantarum* LP26 has limited regulatory effects on metabolic composition, consistent with our previous findings (Guo et al., 2024).

This study demonstrates that the enhanced antioxidant capacity in the C and C_LAB groups was primarily due to the degradation of plant cell wall structures by exogenous cellulase. The significantly reduced NDF and hemicellulose contents in these two treatment groups confirmed that cellulase effectively disrupted mulberry leaf cell walls. This structural breakdown directly promoted the release of bound phenolic compounds, which were originally cross-linked with structural carbohydrates (such as cellulose and hemicellulose) in the cell wall matrix through ester or glycosidic bonds (Pinelo et al., 2006). Direct evidence from this study revealed significant accumulation of phenylpropanoids and polyketides in the cellulase supplemented groups (C and C_LAB), specifically those compounds associated with antioxidant capacity. The number of differentially expressed phenylpropanoids and polyketides in the C vs. CK comparison (80) was substantially higher than in the LAB vs. CK comparison (13) and showed considerable overlap with the C_LAB vs. CK comparison (69). Importantly, 44 metabolites exhibited consistent directional changes and comparable magnitude (with similar FC values) in both the C and C_LAB groups, further confirming that cellulase serves as the key driver of significant alterations in antioxidant-active phenylpropanoids and polyketides in the silage, while the addition of LAB did not significantly enhance this effect.

Although the C and C_LAB groups showed consistent trends for these 44 common differential metabolites, the C group demonstrated stronger antioxidant capacity. This may be attributed to the C group having more uniquely upregulated metabolites (28) and a greater total number of upregulated metabolites (60) (Figures 11C,D), along with specific enrichment of antioxidant-related metabolic pathways such as “Phenylpropanoid biosynthesis” and “Phenylalanine metabolism,” indicating the activation of a broader antioxidant metabolic network. This difference may be due to variations in the microbial community structure between the C and C_LAB groups. The richness and diversity of the bacterial community in the C group were lower than in the C_LAB group. While higher bacterial community richness and diversity are generally unfavorable for silage preservation, they might promote the release of phenolic compounds and enhance the antioxidant capacity of the silage. This is supported by the observation that the LAB group, which had lower bacterial community richness and diversity than the CK group, exhibited lower DPPH and ABTS radical scavenging capacities than the CK group. Certain microorganisms typically considered undesirable in silage, such as yeasts, can promote the biotransformation of phenolic compounds through their metabolic activities (Lachowicz et al., 2017; Martín-Gómez et al., 2021; Kim et al., 2023) Furthermore, the relative abundance of *Pediococcus* in the C group was significantly higher than in the other groups, which may have promoted more complex metabolic network interactions, thereby activating a wider range of antioxidant metabolic pathways. Research has confirmed that African sourdough flatbread fermented with *Pediococcus pentosaceus* shows significantly enhanced antioxidant indices, particularly DPPH radical scavenging activity (Hassan et al., 2024); our previous studies have also confirmed that inoculating mulberry silage with *Pediococcus pentosaceus* alone is more effective in improving antioxidant capacity compared to inoculation with LP26 alone (Guo et al., 2024).

Although the complexity of plant metabolites poses challenges for comprehensively assessing the absolute changes of all metabolites during mulberry silage and their contribution to antioxidant capacity, non-targeted metabolomics, leveraging its advantages of broad-spectrum detection and unbiased screening, enables us to systematically elucidate the mechanisms behind changes in the antioxidant capacity of mulberry silage from the perspective of metabolic dynamics of antioxidant-related phenylpropanoids and polyketides. KEGG pathway enrichment analysis revealed that cellulase treatment significantly affected key metabolic pathways such as “Phenylpropanoid biosynthesis,” “Flavonoid biosynthesis,” and “Isoflavonoid biosynthesis.” Phenylpropanoids are derived from the phenylpropanoid pathway of phenylalanine and tyrosine metabolism (Ohkatsu et al., 2008). The activation of these metabolic pathways directly promotes the biosynthesis and accumulation of secondary metabolites with antioxidant activity. Plants have evolved the ability to synthesize these phenolic compounds as an internal defense mechanism against reactive oxygen species generated under light and heat stress (Ohkatsu et al., 2008). Polyketides are a class of structurally complex and highly diverse natural products, many of which have attracted widespread attention due to their significant biological activities (Zhang and Liu, 2016). The significant accumulation of compounds from these categories detected in the cellulase-treated groups directly explains, at the metabolomic level, the mechanism behind the enhanced antioxidant capacity of the mulberry silage.

Among the co-upregulated compounds in both the C vs. CK and C_LAB vs. CK comparisons, the content of various flavonoids and coumarins known for their strong antioxidant activity significantly increased. For instance, Camelliaside B exhibited the highest VIP values ( > 3.2) in both comparative groups, indicating it is one of the most important signature metabolites distinguishing the treatment groups from the control. This compound, a flavonoid glycoside, is known to be associated with antioxidant activity (Zeng et al., 2024). Quercetin derivatives such as Quercetin 3-O-xylosyl-rutinoside also showed high VIP values ( > 3.0). Quercetin is a recognized potent antioxidant (Jasrotia et al., 2025; Mehta et al., 2025), and the bioavailability and activity of its glycosylated forms have been extensively studied (Chiari-Andréo et al., 2017). Mahaleboside and (7’R,8’R)-4,7’-Epoxy-3’,5-dimethoxy-4’,9,9’-lignanetriol 9’-glucoside exhibited the largest fold changes (FC > 2.7 and > 17, respectively), indicating that cellulase treatment greatly promoted the accumulation or transformation of these compounds. Simultaneously, coumarins (Guerrero et al., 2021), cinnamic acid derivatives (Nouni et al., 2023), and isoflavonoids (Tungmunnithum et al., 2022) were also significantly accumulated in both the C and C_LAB groups. Although this study is constrained by the diversity and complexity of metabolites and did not directly measure the antioxidant capacity of individual phenolic compounds, numerous studies have confirmed that these compounds possess well-defined antioxidant activities. The significant accumulation of these compounds in silage induced by cellulase supplementation is likely the primary driver for the enhanced antioxidant capacity. This finding aligns with and reinforces the conclusions drawn by (Guo et al., 2024), further confirming the crucial role of cellulase in improving the antioxidant capacity of mulberry silage.

Based on the systematic metabolomic evidence obtained in this study, we confirm that cellulase treatment significantly enhances the antioxidant capacity of silage by effectively promoting the release and transformation of phenolic compounds through the degradation of the cell wall structure in mulberry leaves. These findings provide new perspectives for in-depth analysis of the substance transformation mechanisms in mulberry silage. Future research incorporating structural analysis techniques such as Fourier-transform infrared spectroscopy or X-ray diffraction could more directly reveal the alterations in the cell wall’s ultrastructure. This would provide crucial structural biology evidence for elucidating the dissociation mechanism of phenolic-polysaccharide complexes, thereby advancing further investigation into the mechanisms underlying silage quality improvement.

## 6 Conclusion

This study isolated *Lactiplantibacillus plantarum* LP26 from mulberry silage and evaluated its individual and combined effects with cellulase on fermentation. While LP26 improved microbial community structure, it alone had limited impact on phenolic transformation and antioxidant capacity. In contrast, cellulase significantly enhanced fermentation quality by reducing pH and ammonia nitrogen while increasing LA and water-soluble carbohydrates through degradation of fiber components. More importantly, it released bound phenolics and promoted their conversion into antioxidants like flavonoids and coumarins, markedly boosting antioxidant activity. Although combining LAB and cellulase synergistically increased Lactobacillus abundance, it did not provide additive benefits in fiber degradation, phenolic conversion, or antioxidant enhancement, indicating that cellulase is the key driver for improving antioxidant functionality in mulberry silage.

## Data Availability

The sequencing data were deposited in the Sequence Read Archive (SRA) under the accession number of PRJNA1328713.

## Footnotes

1
https://www.ncbi.nlm.nih.gov/

## Supplementary material

Supplementary Figure 1 Comparative profiling of phenylpropanoids and polyketides in C vs CK comparison. (A) Overview of metabolite variants. (B) Classification at the class level. (C) Classification at the sub-class level.

Supplementary Figure 2 Comparative profiling of phenylpropanoids and polyketides in C vs LAB comparison. (A) Overview of metabolite variants. (B) Classification at the class level. (C) Classification at the subclass level.

Supplementary Figure 3 Comparative profiling of phenylpropanoids and polyketides in C vs C_LAB comparison. (A) Overview of metabolite variants. (B) Classification at the class level. (C) Classification at the subclass level.
