# Supplementary material for: Cellulase over lactic acid bacteria in enhancing antioxidant capacity of mulberry silage via phenolic release
Source: Front Microbiol. 2025 Dec 11;16:1725406. doi: 10.3389/fmicb.2025.1725406 (PMC12738817; doi:10.3389/fmicb.2025.1725406)
Supplement: Supplementary file 1 [file Table_1.docx]

Appendix A

**Table A1** VIP scores and fold-change values for 45 shared phenylpropanoids and polyketides in the C and C_LAB groups relative to the CK group

| No. | phenylpropanoids and polyketide | VIP | | FC | |
| --- | --- | --- | --- | --- | --- |
|  |  | C | C_LAB | C | C_LAB |
| 1 | (7'R,8'R)-4,7'-Epoxy-3',5-dimethoxy-4',9,9'-lignanetriol9'-glucoside | 1.48 | 1.63 | 0.87 | 0.86 |
| 2 | (7'R,8'R)-4,7'-Epoxy-3'-methoxy-4',5,9,9'-lignanetetrol9'-glucoside | 2.11 | 2.03 | 17.17 | 19.04 |
| 3 | 1-(2,6-Dihydroxy-4-methoxyphenyl)-3-phenyl-1-propanone | 1.63 | 1.68 | 0.84 | 0.82 |
| 4 | 2-Butyl-3-phenyl-2-propen-1-al | 1.84 | 1.54 | 1.16 | 1.11 |
| 5 | 2''-O-Acetylisoorientin | 1.64 | 1.88 | 0.79 | 0.77 |
| 6 | 3Hydroxycoumarin | 1.23 | 1.29 | 1.07 | 1.07 |
| 7 | 3'-Hydroxy-4',5,6,7,8-pentamethoxyflavone | 1.24 | 1.35 | 1.10 | 1.11 |
| 8 | 3-Hydroxy-4-methoxyphenyllacticacid | 1.11 | 1.24 | 1.08 | 1.10 |
| 9 | 4-Coumaroylputrescine | 2.20 | 2.09 | 1.39 | 1.36 |
| 10 | 4-Hydroxycinnamoylagmatine | 2.47 | 2.21 | 1.76 | 1.61 |
| 11 | 4-Methylumbelliferoneglucuronide | 1.47 | 1.85 | 0.86 | 0.80 |
| 12 | Caffeicacidethylester | 1.12 | 1.22 | 1.12 | 1.13 |
| 13 | CamelliasideB | 3.27 | 3.28 | 2.13 | 2.12 |
| 14 | Cyclocurcumin | 1.55 | 1.01 | 1.24 | 1.11 |
| 15 | Glechomafuran | 1.53 | 1.21 | 1.14 | 1.08 |
| 16 | Glycitein | 1.63 | 1.66 | 1.22 | 1.21 |
| 17 | Glycitin | 2.08 | 1.86 | 1.46 | 1.38 |
| 18 | Gravolenicacid | 1.58 | 1.61 | 1.18 | 1.18 |
| 19 | Irisolidone7-O-glucuronide | 1.57 | 1.67 | 0.81 | 0.81 |
| 20 | Isopulegonecaffeate | 1.99 | 2.08 | 1.30 | 1.32 |
| 21 | Isoquercitrin | 2.48 | 2.51 | 1.34 | 1.34 |
| 22 | Isowertin2''-rhamnoside | 2.04 | 1.81 | 1.27 | 1.22 |
| 23 | Loquatoside | 2.77 | 2.60 | 1.84 | 1.74 |
| 24 | Mahaleboside | 2.65 | 2.82 | 2.77 | 2.94 |
| 25 | Mono-trans-p-coumaroylmesotartaricacid | 1.07 | 1.01 | 1.06 | 1.05 |
| 26 | Morin | 2.35 | 2.38 | 1.25 | 1.26 |
| 27 | Myricetin | 1.41 | 1.52 | 0.90 | 0.89 |
| 28 | N-(p-Hydroxyphenyl)ethylp-hydroxycinnamide | 2.28 | 2.19 | 0.76 | 0.78 |
| 29 | Nb-p-Coumaroyltryptamine | 2.26 | 1.27 | 1.33 | 1.15 |
| 30 | N-cis-Caffeoyltyramine | 2.14 | 1.93 | 0.75 | 0.80 |

**Table S1.** Continued.

| No. | phenylpropanoids and polyketide | VIP | | FC | |
| --- | --- | --- | --- | --- | --- |
|  |  | C | C_LAB | C | C_LAB |
| 31 | N'-Hydroxyneosaxitoxin | 2.35 | 1.88 | 1.69 | 1.32 |
| 32 | Osthenol | 1.33 | 1.51 | 0.89 | 1.10 |
| 33 | Quercetin3-(2Gal-apiosylrobinobioside) | 3.05 | 1.17 | 1.48 | 1.46 |
| 34 | Quercetin3,4'-Diglucoside | 1.88 | 2.99 | 0.82 | 0.81 |
| 35 | Quercetin3-galactoside | 1.19 | 1.89 | 0.90 | 0.85 |
| 36 | Quercetin3-O-glucosyl-xyloside | 1.70 | 1.54 | 0.90 | 0.88 |
| 37 | Quercetin3-O-xylosyl-rutinoside | 3.13 | 3.15 | 1.58 | 1.58 |
| 38 | Rutaretin9-rutinoside | 1.50 | 1.25 | 1.49 | 1.40 |
| 39 | Subaphylline | 2.57 | 1.78 | 0.59 | 0.75 |
| 40 | Tetracycline | 2.81 | 2.49 | 1.67 | 1.55 |
| 41 | Tulipanin | 2.75 | 2.77 | 1.42 | 1.42 |
| 42 | Umbelliferone | 2.28 | 2.37 | 1.34 | 1.34 |
| 43 | Xanthomicrol | 1.99 | 2.22 | 1.25 | 1.29 |
| 44 | Yangonin | 1.17 | 1.22 | 1.11 | 1.12 |
| 45 | Zearalenone4-sulfate | 1.42 | 1.81 | 1.24 | 1.37 |
