# Supplementary figures and images for: Cellulase over lactic acid bacteria in enhancing antioxidant capacity of mulberry silage via phenolic release
Source: Front Microbiol. 2025 Dec 11;16:1725406. doi: 10.3389/fmicb.2025.1725406 (PMC12738817; doi:10.3389/fmicb.2025.1725406)

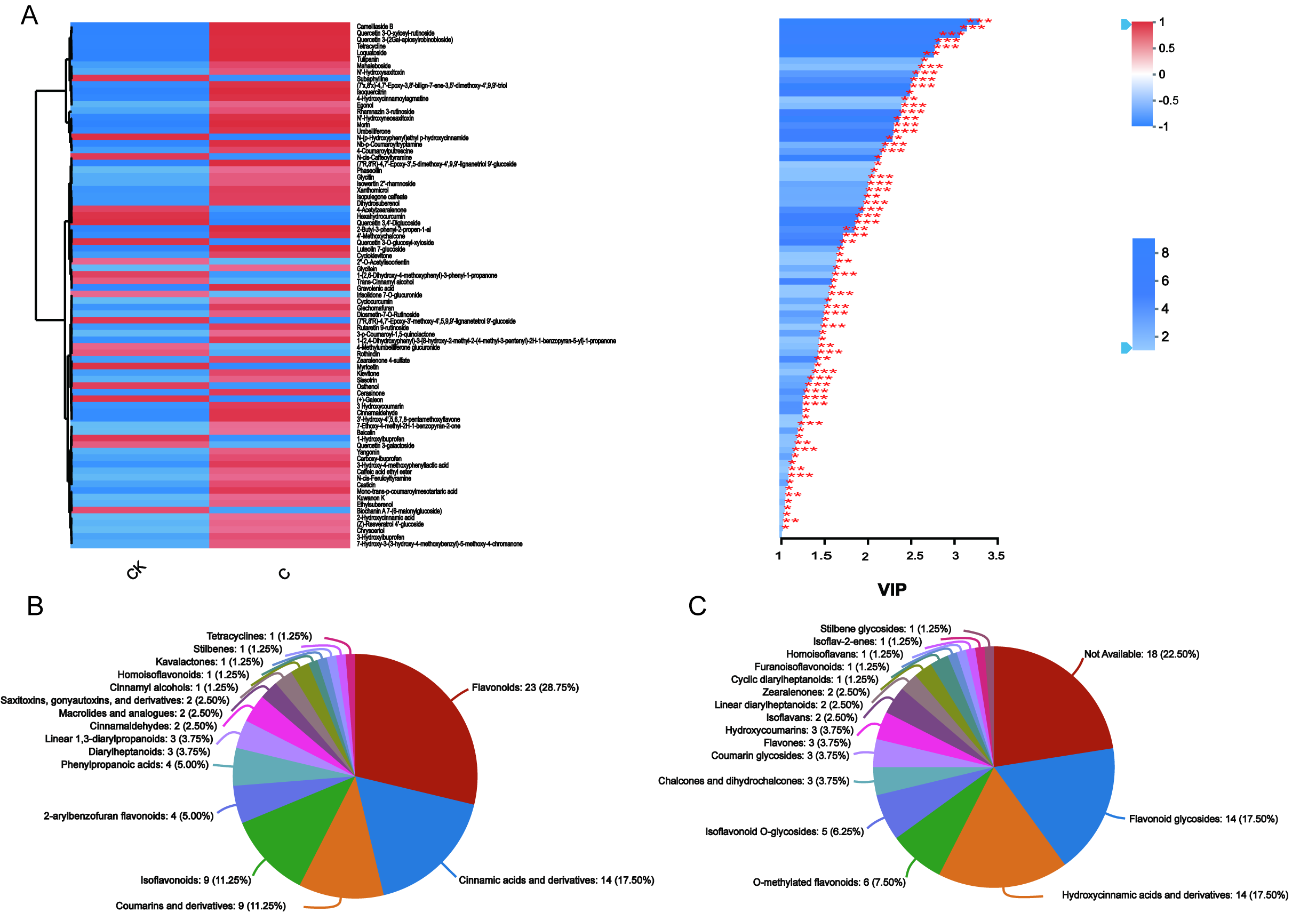

Supplement: Supplementary Figure 1 — Comparative profiling of phenylpropanoids and polyketides in C vs CK comparison. (A) Overview of metabolite variants. (B) Classification at the class level. (C) Classification at the sub-class level. [file Image_1.tif]

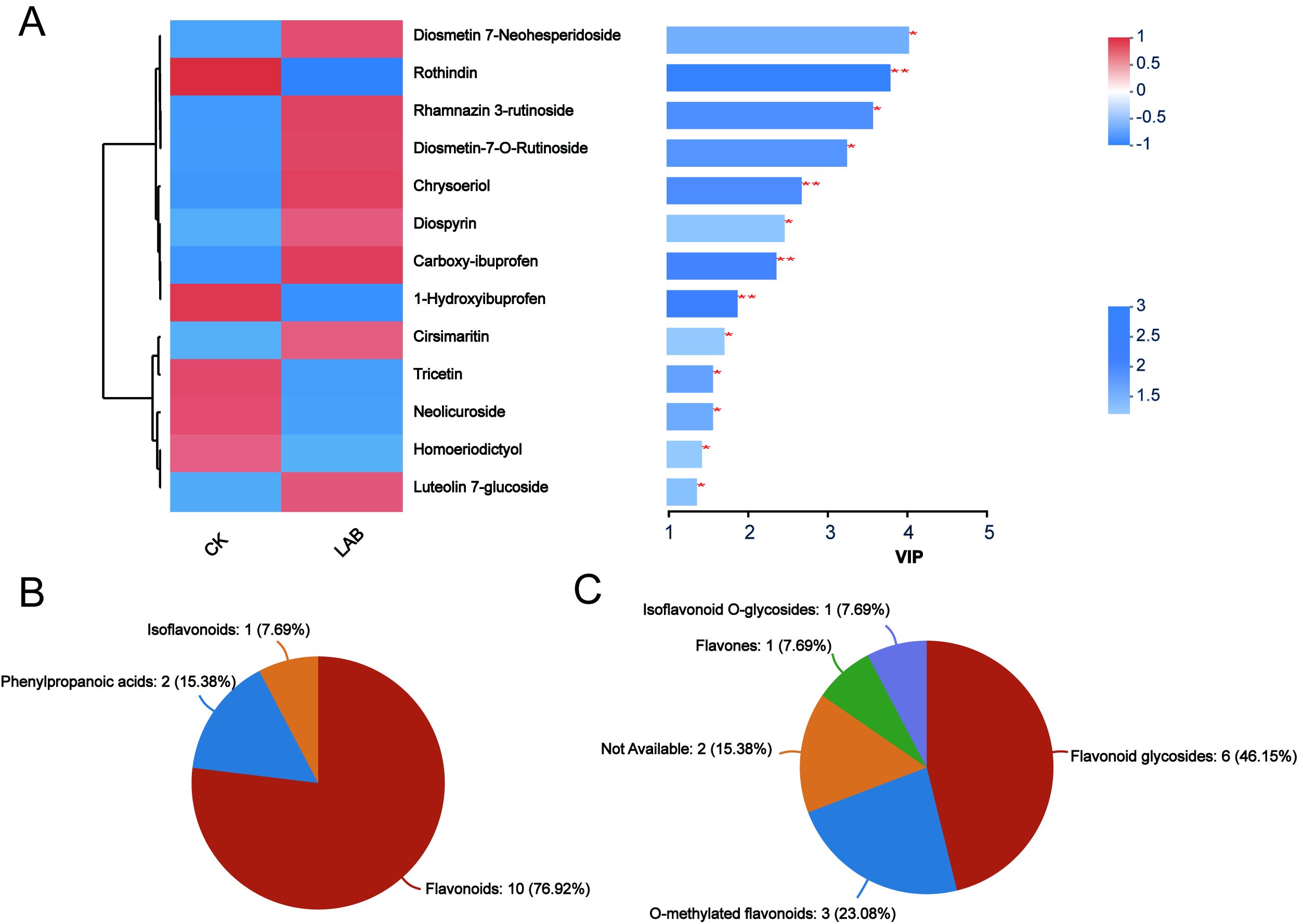

Supplement: Supplementary Figure 2 — Comparative profiling of phenylpropanoids and polyketides in C vs LAB comparison. (A) Overview of metabolite variants. (B) Classification at the class level. (C) Classification at the subclass level. [file Image_2.tif]

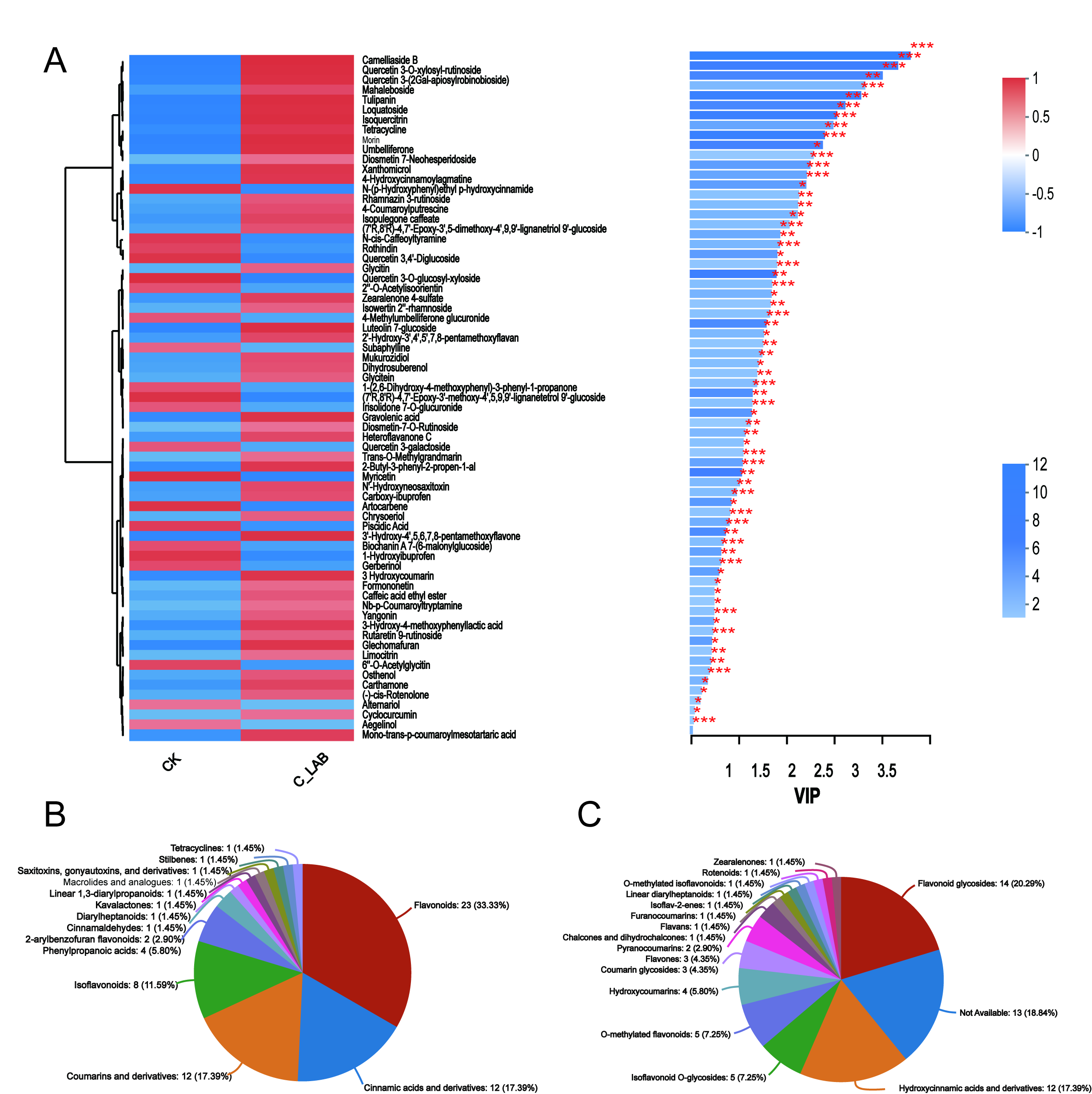

Supplement: Supplementary Figure 3 — Comparative profiling of phenylpropanoids and polyketides in C vs C_LAB comparison. (A) Overview of metabolite variants. (B) Classification at the class level. (C) Classification at the subclass level. [file Image_3.tif]
